# Lipopolyplex-formulated mRNA cancer vaccine elicits strong neoantigen-specific T cell responses and antitumor activity

**DOI:** 10.1126/sciadv.adn9961

**Published:** 2024-10-11

**Authors:** Ting Fan, Congcong Xu, Jichuan Wu, Yihua Cai, Wanlu Cao, Haifa Shen, Mingna Zhang, Hanfei Zhu, Jingxian Yang, Zhounan Zhu, Xiaopin Ma, Jiale Ren, Lei Huang, Qianyun Li, Yuying Tang, Bo Yu, Chunxiu Chen, Mingcheng Xu, Qiuhe Wang, Zhuya Xu, Fengjia Chen, Shujing Liang, Zhixian Zhong, Anmbreen Jamroze, Dean G. Tang, Hangwen Li, Chunyan Dong

**Affiliations:** ^1^ Department of Oncology, Shanghai East Hospital, School of Medicine, Tongji University, Shanghai 200092, China.; ^2^ StemiRNA Therapeutics Inc., Shanghai, China.; ^3^ Biomedical Polymers Laboratory, College of Chemistry Chemical Engineering and Materials Science and State Key Laboratory of Radiation Medicine and Protection, Soochow University, Suzhou 215123, China.; ^4^ International College of Pharmaceutical Innovation, Soochow University, Suzhou 215123, China.; ^5^ College of Pharmaceutical Sciences, Soochow University, Suzhou 215123, China.; ^6^ Department of Pharmacology & Therapeutics and Experimental Therapeutics (ET) Graduate Program, University at Buffalo and Roswell Park Comprehensive Cancer Center, Buffalo, NY 14263, USA.

## Abstract

mRNA neoantigen cancer vaccine inducing neoantigen-specific T cell responses holds great promise for cancer immunotherapy; however, its clinical translation remains challenging because of suboptimal neoantigen prediction accuracy and low delivery efficiency, which compromise the in vivo therapeutic efficacy. We present a lipopolyplex (LPP)–formulated mRNA cancer vaccine encoding tandem neoantigens as a cancer therapeutic regimen. The LPP-formulated mRNA vaccines elicited robust neoantigen-specific CD8^+^ T cell responses in three syngeneic murine tumor models (CT26, MC38, and B16F10) to suppress tumor growth. Prophylactic cancer vaccine treatment completely prevented tumor development, and long-lasting memory T cells protected mice from tumor cell rechallenge. Combining the vaccine with immune checkpoint inhibitor further boosted the antitumor activity. Of note, LPP-based personalized cancer vaccine was administered in two cancer patients and induced meaningful neoantigen-specific T cell and clinical responses. In conclusion, we demonstrated that the LPP-based mRNA vaccine can elicit strong antitumor immune responses, and the results support further clinical evaluation of the therapeutic mRNA cancer vaccine.

## Teaser

LPP encapsulated mRNA-based neoantigen cancer vaccine elicits neoantigen-specific immunity.

## INTRODUCTION

Neoantigens arising from mutations in cancer cells are important targets for T cell–mediated immunity (*1*). Compared to other antigen types such as tumor-associated antigens, neoantigens have the distinct advantage as ideal targets for effective personalized cancer therapy owing to their tumor specificity (*2*). They are highly antigenic as to induce robust tumor-specific T cell responses (*3*). With the availability of next-generation sequencing and bioinformatics tools, rapid identification of gene mutations and prediction of neoantigens have become possible (*4*). It is anticipated that more neoantigen-based vaccines will be developed for personalized cancer therapy. Multiple forms of cancer vaccines have been evaluated in preclinical animal models and clinical trials, including peptide, DNA, and dendritic cell (DC)–based vaccines. However, the anticancer efficacy of these vaccines remains to be improved in general (*5*). With the success of prophylactic vaccines for SARS-CoV-2 (*6*–*9*), mRNA-based cancer vaccine has gained broad attention recently (*10*). As naked mRNA molecules have poor cell permeability and are susceptible to degradation, different delivery platforms have been exploited to develop mRNA-based personalized cancer vaccine (PCV) including lipid nanoparticles (*11*) and lipoplexes (*12*). Preclinical and clinical studies have revealed that mRNA vaccines tend to elicit T helper 1 (T_H_1)–biased immune responses, and both the antigen-encoding mRNA molecule and the excipients in the packaging vehicle may be responsible for the adjuvant activity (*13*, *14*). Nevertheless, T_H_1-biased cellular and humoral responses are a prerequisite for potent antitumor immunity by PCV (*15*–*18*). Despite these advances, challenges remain in accurate prediction of neoantigens, efficient in vivo delivery of antigen-encoded mRNA, and prolonged mRNA expression.

To stimulate potent antitumor immune responses, it is essential to identify high-quality neoantigens and to ensure high-level sustained expression of the antigenic peptide (*19*–*22*). We have developed a proprietary SmartNeo platform to accurately identify nonsynonymous mutant variants (including single nucleotide variants and insertion-deletion), analyze tumor mutation characteristics, quantify gene/transcript expression, and perform human leukocyte antigen (HLA) typing. SmartNeo surpasses the 43% threshold in our investigative work, a performance metric that outperforms other publicly accessible neoantigen screening algorithms (*15*, *23*). In addition, we have recently reported an algorithm, termed LinearDesign, for mRNA design to improve the stability of mRNA for enhanced protein expression (*24*). The optimized mRNA molecules are then formulated in the proprietary lipopolyplex (LPP) delivery vehicle (*25*), which has been demonstrated in the development of mRNA vaccines for COVID-19 and other infectious diseases (*26*–*29*). The core-shell structure of LPP protected the encapsulated mRNA from rapid degradation in vivo and efficiently delivered mRNA to antigen-presenting cells (APCs). The LPP-based mRNA vaccine demonstrated a good safety profile with potent efficacy in preclinical studies (*27*) and clinical trials (*29*).

**Fig. 1.**
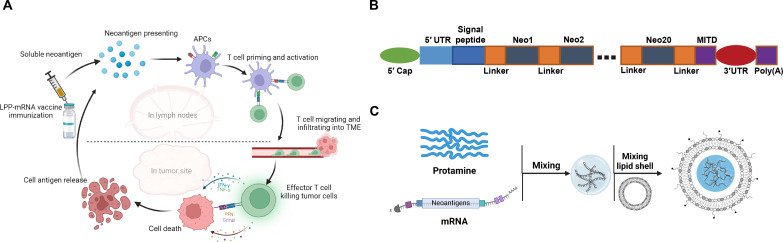
The schematic illustrates an LPP-formulated mRNA PCV. (**A**). Diagram of the immune mechanisms of LPP-mRNA vaccine in vivo. (**B**). Schematic presentation of the neoantigen-coding mRNA sequences in the LPP-mRNA vaccine. (**C**). Schematic depiction of the steps in preparing the LPP-mRNA vaccine.

In this study, we applied LPP to encapsulate neoantigen-encoding mRNA molecules as a PCV and evaluated its immunogenicity and efficacy in murine tumor models (Fig. 1A). Neoantigen sequences predicted by SmartNeo algorithm were tandemly linked to form a polypeptide. The LinearDesign algorithm was then applied to optimize the mRNA sequence without altering the encoded polypeptide sequence. Three murine models (CT26, MC38, and B16F10) were used to evaluate the efficacy of PCV. Mice with tumor remission were rechallenged with tumor cell inoculation to test T memory cell activities. Furthermore, combination therapy of mRNA vaccine with immune checkpoint inhibitor (ICI) antibodies was adopted to sensitize tumor against antigen-specific T cell immune responses. Last, we also monitored the in vivo immune response of patients after the administration of LPP-PCV and evaluated the potential clinical benefits with the goal of further clinical applications in the future.

## RESULTS

### Preparation and characterizations of LPP-based mRNA vaccine

We performed whole-exosome sequencing (WES) and mRNA sequencing of murine tumor cell lines to identify tumor-specific mutations and applied SmartNeo to predict potential immunogenic neoantigens (table S1). The identified candidate neoantigens were then aligned in a linear tandem form separated by spacers. A major histocompatibility complex (MHC) class I trafficking signal (MITD) sequence was inserted at the tail end to enhance intracellular trafficking of expressed neoantigens to the MHC complex (*30*, *31*). Then, 5′ untranslated region (5′UTR) and 3′UTRs were added to complete the mRNA sequence (Fig. 1B).

A two-step microfluidics-based process was used to prepare LPP-based mRNA vaccine nanoparticles (Fig. 1C). Transmission electron microscopy (TEM) revealed the core-shell structure of LPP-based nanoparticles, showing mRNA polyplex cores encased by the lipid shell, with an average particle size of 100 nm (fig. S1A). LPP-mRNA nanoparticles were stable during storage at 4° or −20°C over 4 weeks based on particle size and zeta potential measurement (fig. S1B). LPP-mRNA was very efficient in transfecting 293T cells based on flow cytometry and fluorescent microscopic analyses using the mRNA encoding enhanced green fluorescent protein (GFP) as a surrogate (fig. S2, A to C). In addition, LPP-mRNA did not show cytotoxicity when applied to 293T cells, even at high concentrations (fig. S2D).

We assessed the biodistribution of the LPP-mRNA vaccine in Institute of Cancer Research (ICR) mice after a single dose of subcutaneous inoculation, and gene copy numbers in tissues were determined with quantitative polymerase chain reaction. We observed that the mRNA concentration reached peak level at 6 hours in the circulation, and the spleen and draining lymph nodes had the highest concentration (fig. S2E). Since the spleen and lymph nodes are the primary tissues for immune cells, the biodistribution pattern favors immune responses after vaccine inoculation. In addition, the MITD protein could be detected in cell lysates after coincubation of 293T cells with the LPP-mRNA vaccine, confirming that the mRNA sequence could be successfully translated into neoantigen epitopes in cells (fig. S2F).

### LPP-mRNA vaccine induces antigen presentation and T cell activation

**Fig. 2.**
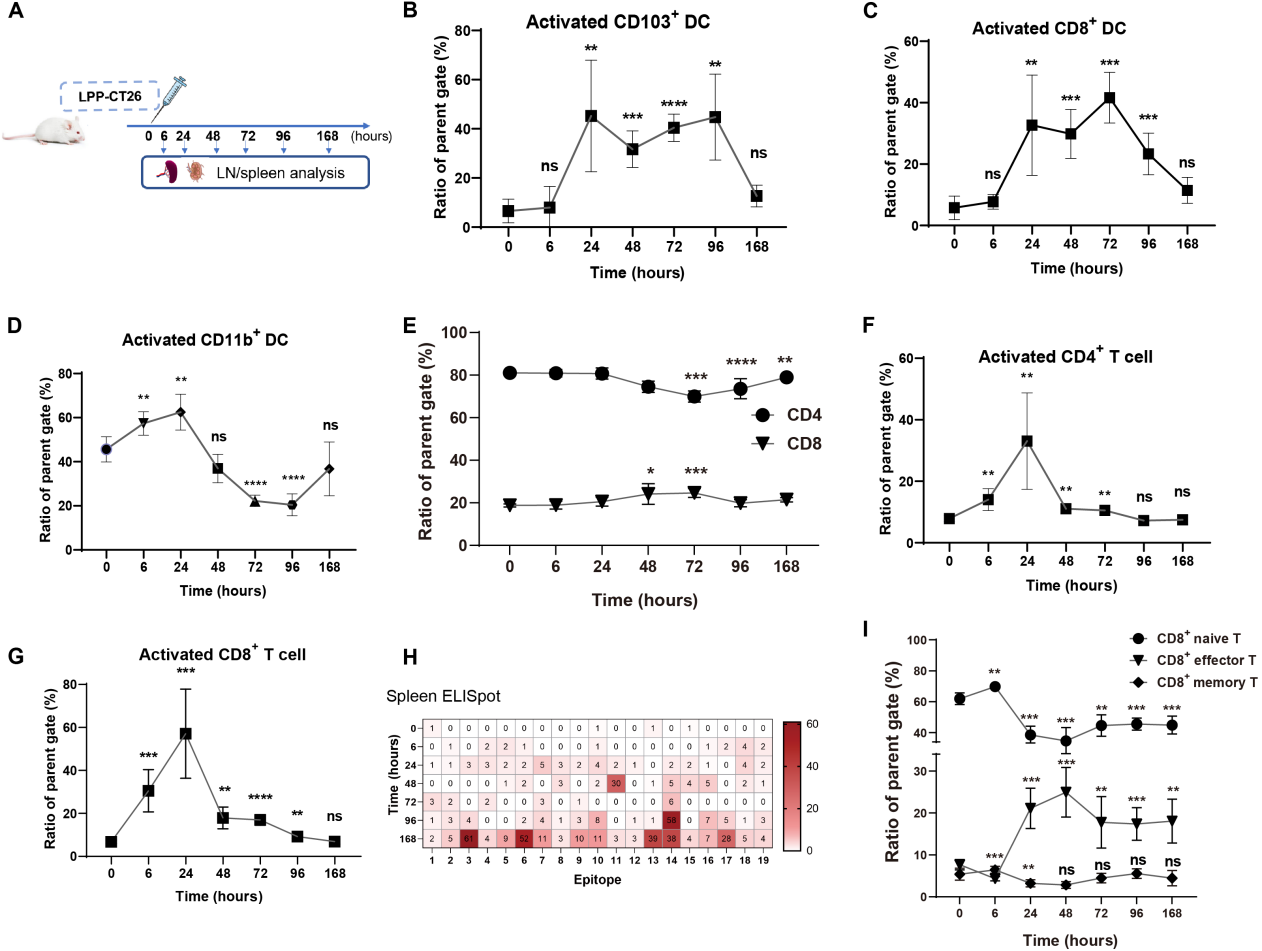
LPP-CT26 induces DC activation, antigen presentation, and T cell activation in vivo. (**A**). Treatment schedule for LPP-CT26 vaccination (*n* = 5 mice per group) and correlative immune analysis. LN, lymph nodes. (**B** to **D**). Analysis of activation of DC subsets in lymph nodes after LPP-CT26 treatment. (**E**). Proportion of total CD4^+^ and CD8^+^ T cells in lymph nodes after LPP-CT26 immunization. (**F** and **G**). Proportion of CD4^+^ and CD8^+^ T cell activation in lymph nodes after LPP-CT26 immunization. (**H**). ELISpot assay of neoantigen-induced T cell (splenocyte) activation at the indicated time points. (**I**). Relative proportion of effector and memory T cells in lymph nodes at different time points upon LPP-CT26 vaccination. Data represent the means ± SD. One-way analysis of variance (ANOVA) test was performed for all comparisons. (*P < 0.05, **P < 0.01, ***P < 0.001, ****P < 0.0001). ns, not significant.

We treated BALB/c mice with CT26-specific LPP-mRNA vaccine (LPP-CT26) and monitored longitudinal immune response at various time points to assess immunogenicity from the vaccine (Fig. 2A). The mRNA sequences in LPP-CT26 were tailored on the basis of gene mutations and expression level of the mutant genes in the CT26 tumor cells. Activation and antigen presentation capability of DCs in lymph nodes were assessed (Fig. 2 and fig. S3). Within 24 hours of vaccination, conventional type I DCs (cDC1) exhibited arresting maturation, a response that persisted for 72 to 96 hours (Fig. 2, B and C). This maturation was primarily observed in CD8^+^ DCs and CD103^+^ DCs (cDC1s) (Fig. 2, B and C), which are known for their roles in cross-presenting antigens to MHC class I and facilitating effective CD8^+^ T cell activation in tumor-draining lymph nodes and tumors (*32*). In contrast, the abundance of CD11b^+^ cDC2s slightly increased within 24 hours of immunization but then declined (Fig. 2D). Subsequently, we analyzed changes and activation of different T cell subsets in immunized mice (Fig. 2, E to I, and figs. S4 to S6). The percentage of total CD8^+^ (but not CD4^+^) T cells in lymph nodes and spleen increased slightly within 48 to 72 hours after vaccination (Fig. 2E and fig. S4A). LPP-CT26–treated mice displayed the activation of CD4^+^ and CD8^+^ T cells rather quickly and transiently, i.e., within 6 to 24 hours, indicating rapid immune activation by LPP-formulated cancer vaccine in vivo (Fig. 2, F and G, and fig. S4, B and C). Immune responses in the spleen corresponding to each neoantigen were then analyzed following LPP-CT26 immunization, and we observed that splenocytes showed a time-dependent increase in interferon-γ (IFN-γ) production in response to individual neoantigens indicating effective activation of neoantigen-specific T cells (Fig. 2H). The SmartNeo algorithm used to predict the LPP-CT26 neoantigen response demonstrated an experimental precision rate of 52.6% (10 of 19) (Fig. 2H). Furthermore, the percentage of effector T cells remarkably increased within 24 hours and persisted in both lymph nodes and splenocytes (Fig. 2I and fig. S4, D to F). This rapid increase in the effector T cell population would support the enhanced antitumor activity (see below). Together, these findings demonstrate that the LPP-based mRNA vaccine can efficiently prime and amplify the population of neoantigen-specific T cells, a critical step for mounting a robust antitumor immune response.

### Optimizing LPP-mRNA vaccine administration leads to better antitumor efficacy

**Fig. 3.**
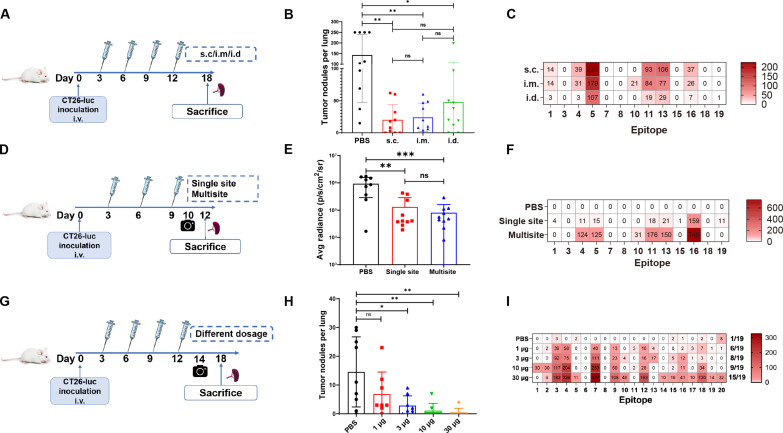
Optimizing of LPP-mRNA vaccination procedures improved anti-tumor efficacy. (**A** to **C**). Effects of LPP-mRNA vaccination routes on immune response and experimental lung metastasis. Shown are experimental schema for delivering the LPP-CT26 vaccine via three different administration routes (*n* = 10 mice per route) (A); bar graphs presenting the end point (day 18) lung metastases in mice that received intravenously injected CT26-luc cells and LPP-CT26 vaccine administered via three different routes (B); and IFN-γ ELISpot assay of splenocytes from mice inoculated with the LPP-CT26 vaccine delivered via three routes (C). s.c., subcutaneous; i.m., intramuscular; i.d., intradermal; i.v., intravenous. (**D** to **F**). Effects of single- versus multisite LPP-mRNA vaccination on immune response and tumor burden. Shown are experimental schema with single or multiple vaccination sites (*n* = 10 mice per group) (D); bar graphs presenting relative tumor burden assessed by fluorescent intensity of CT26-luc tumors in mice on day 10 (E); and IFN-γ ELISpot assay in splenocytes from mice with single or multisite LPP-CT26 vaccinations (F). (**G** to **I**). Effects of LPP-mRNA vaccine dosage on immune response and lung metastasis. Shown are experimental schema (*n* = 10 mice per dose) (G); bar graphs presenting lung metastases in different LPP-CT26 dosage groups (H); and IFN-γ ELISpot assay of splenocytes in different LPP-CT26 dosage groups (I). Data were presented as means ± SD. One-way ANOVA statistical test was performed was performed for all data analysis. (*P < 0.05, **P < 0.01, ***P < 0.001).

The CT26 syngeneic mouse tumor is a classical model for tumor antigen exploration, as its somatic mutations provide tumor-specific T cell targets (*33*). Following sequencing and algorithmic prediction of luciferase-labeled CT26 cells (CT26-luc), we identified the top 20 candidate immunogenic neoantigens for the design and synthesis of LPP-CT26. To find the optimal administration route for vaccination, we evaluated the antitumor effect and immunogenicity of intravenous, intramuscular, and intradermal injections of LPP-CT26 in a CT26-luc lung metastasis mouse model (Fig. 3A). The results showed that the subcutaneous group exhibited fewer pulmonary metastatic nodules compared to the intramuscular and intramuscular groups, suggesting a more effective antitumor response by the subcutaneous route (Fig. 3B and fig. S7A). In addition, the subcutaneous group demonstrated higher secretion of IFN-γ against each neoantigen, as determined by enzyme-linked immunospot (ELISpot) assay (Fig. 3C). These results indicated a robust neoantigen-specific T cell response to LPP-CT26 administered via the subcutaneous route.

Next, we investigated the effects of frequency and dosing concentrations of LPP-CT26 and found that its antitumor efficacy was improved by increasing administration sites while keeping the LPP-CT26 dose constant (Fig. 3, D and E, and fig. S7, B and C). The response rate and intensity of neoantigens were both elevated in the multisite injection group (Fig. 3F), further supporting the advantages of this approach. Moreover, we also observed a dose-dependent inhibitory effect of LPP-CT26 on pulmonary metastatic load when the vaccine was given through multisite injections (Fig. 3, G and H, and fig. S7D). The IFN-γ ELISpot assays demonstrated that 15 of the 19 evaluable neoantigens induced a T cell response in the LPP-CT26 30-μg group, which was higher than that in lower dosage groups (Fig. 3I). These results underscored the importance of optimizing the vaccine dosage and administration method to achieve favorable antitumor effects.

### LPP-mRNA vaccine controls tumor growth by neoantigen-specific immune response

**Fig. 4.**
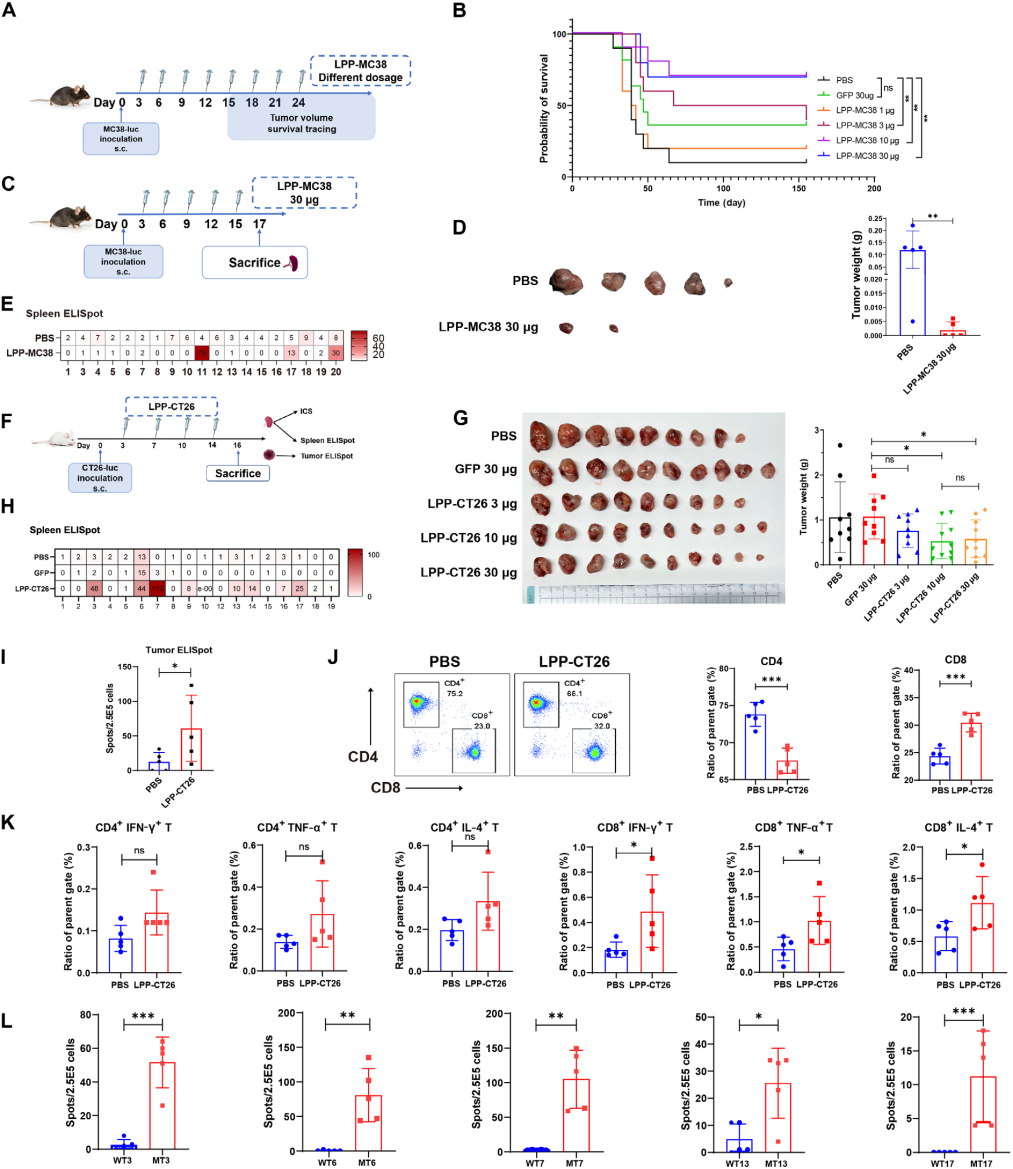
LPP-mRNA vaccine elicits neoantigen-specific immune responses to control tumor growth in syngeneic tumor models. (**A**) Experimental schema to assess the antitumor activity of LPP-MC38 in the MC38 tumor model. C57BL/6 mice (*n* = 10 per group) were subcutaneously inoculated with MC38-luc tumor cells and then immunized with different doses of LPP-MC38 vaccines. (**B**) Survival rate in mice (*n* = 10 mice per group) receiving eight immunizations with different doses of LPP-MC38 or irrelevant (GFP) LPP. (**C** to **E**) Effects of LPP-MC38 on primary tumor growth and the immune response. MC38-luc tumor cells were subcutaneously implanted in the left flanks of mice, and 3 days later, the mice were immunized with LPP-MC38 (*n* = 5) or PBS (*n* = 5) for a total of five injections (C). The end point (day 17) tumor images and weights were presented (D). IFN-γ ELISpot assay in splenocytes restimulated with the individual peptides (E). (**F** to **I**) Effects of LPP-CT26 on primary tumor growth and the immune response. Experimental schema to assess the antitumor activity of LPP-CT26 in BALB/c (*n* = 10 per group) mice bearing subcutaneous CT26-luc tumors immunized with different doses of LPP-CT26 or irrelevant (GFP) LPP or PBS (F). Shown in (G) are the end point (day 16) CT26-luc tumor images and weights (means ± SD). Shown in (H) and (I) are IFN-γ ELISpot assays of splenocytes in CT26-bearing mice (H) and for a representative neoantigen in CT26 tumor tissue [*n* = 5 (I)]. (**J**) Flow cytometric analysis of CD4^+^ and CD8^+^ T cells in bulk splenocytes. (**K**) ICS by fluorescence-activated cell sorting (FACS) in T cells in mouse bulk splenocytes treated with LPP-CT26 or PBS. (**L**) IFN-γ ELISpot assays for immunogenic neoantigens and their corresponding WT sequences. Data were presented as means ± SD. One-way ANOVA statistical test was performed for all data analysis. (*P < 0.05, **P < 0.01, ***P < 0.001).

We subsequently determined whether our LPP-mRNA vaccine had tumor-controlling effects and investigated the role of specific T cell subsets and the underlying mechanisms in the process. To this end, we used the MC38, a colorectal cancer cell line with high microsatellite instability, as the tumor model (Fig. 4A). We observed a dose-dependent tumor-inhibitory effect of LPP-MC38 vaccine. At the 6-month follow-up, 7 of 10 mice in each of the 10- and 30-μg LPP-MC38 groups achieved long-term disease-free survival, notably surpassing the survival rate of the phosphate-buffered saline (PBS) group (1 of 10) and the scramble (GFP) LPP control group (4 of 10) (Fig. 4B). Consistent with the survival data, robust tumor suppression was evident in the 30-μg LPP-MC38 group on day 17 (Fig. 4, C and D). When the splenocytes were restimulated with neoepitopes corresponding to LPP-MC38, 3 of 20 neoantigens elicited specific IFN-γ ELISpot responses in the LPP-MC38 group. In contrast, no such responses were detected in the control group (Fig. 4E). The impressive tumor-suppressive effects of LPP-MC38 highlighted the potential of the LPP-mRNA vaccine platform.

We performed similar tumor and mechanistic studies in mice bearing subcutaneous CT26-luc tumors that received multiple LPP-CT26 vaccinations (Fig. 4F). We observed that LPP-CT26, at both 10 and 30 μg, reduced tumor weight by about 50% when compared to LPP-GFP at 30 μg (Fig. 4G). On the other hand, we observed that although immunization with LPP-CT26 can moderately increase the proportion of tumor-infiltrating lymphocytes (TILs), the levels remain relatively limited (fig. S8, A and B). This phenomenon might be why LPP-CT26 failed to elicit more prominent inhibitory effects in the subcutaneous CT26 tumor model. Neoantigen-specific immune responses were observed when splenocytes were restimulated with CT26 neoepitopes (Fig. 4H), and a neoantigen-specific immune response against #7 epitope of TILs in the tumor tissue was also detected by IFN-γ ELISpot assay (Fig. 4I). These findings suggest that neoantigen-specific T cells were not only activated by LPP-CT26 vaccine but also actively migrated and infiltrated into the tumor microenvironment, contributing to tumor control.

Epitope recognition represents a crucial step in staging effective antitumor immune responses. We observed a notable increase in splenic CD8^+^ T cells following LPP-CT26 vaccination in comparison to the control, while the ratio of CD4^+^ T cells decreased (Fig. 4J). To investigate the functional aspects of T cell responses induced by LPP-mRNA immunization, splenocytes were restimulated with neoantigens encoded by LPP-CT26, and flow cytometry was used to identify intracellular cytokines produced by both CD4^+^ and CD8^+^ T cells (fig. S9). Intracellular cytokine staining (ICS) analysis revealed increased percentages of IFN-γ+, tumor necrosis factor–α^+^ (TNF-α^+^), and interleukin-4^+^ (IL-4^+^) CD8^+^ T cells and a trend (though no statistical significance) of IFN-γ^+^, TNF-α^+^, and IL-4^+^ CD4^+^ T cells with LPP-CT26 neoantigen stimulation (Fig. 4K). There were no detectable differences in IL-2^+^ CD8^+^ or CD4^+^ T cells (fig. S10). Notably, the frequency of neoantigen-responsive CD8^+^ T cells appeared to be higher than CD4^+^ T cells (Fig. 4K), potentially underscoring the crucial role of CD8^+^ T cells in exerting antitumor activities.

We restimulated the splenocytes with the five neoantigens that exhibited reactivity in LPP-CT26 and their corresponding wild-type (WT) peptides. Our findings revealed that all five neoantigen peptides from LPP-CT26 demonstrated immunogenicity, as determined by the IFN-γ ELISpot assay, whereas the corresponding WT peptides failed to elicit any discernible immune response (Fig. 4L). These results affirm that neoantigens have a distinct propensity for immune recognition with minimal cross-reactivity between neoantigens and WT peptides. Collectively, the preceding data suggest that the robust antitumor efficacy observed following LPP-mRNA vaccination is intricately linked to the induction of neoantigen-specific immune responses.

### CD8^+^ T cell–mediated immune response effectively contributes to tumor control

**Fig. 5.**
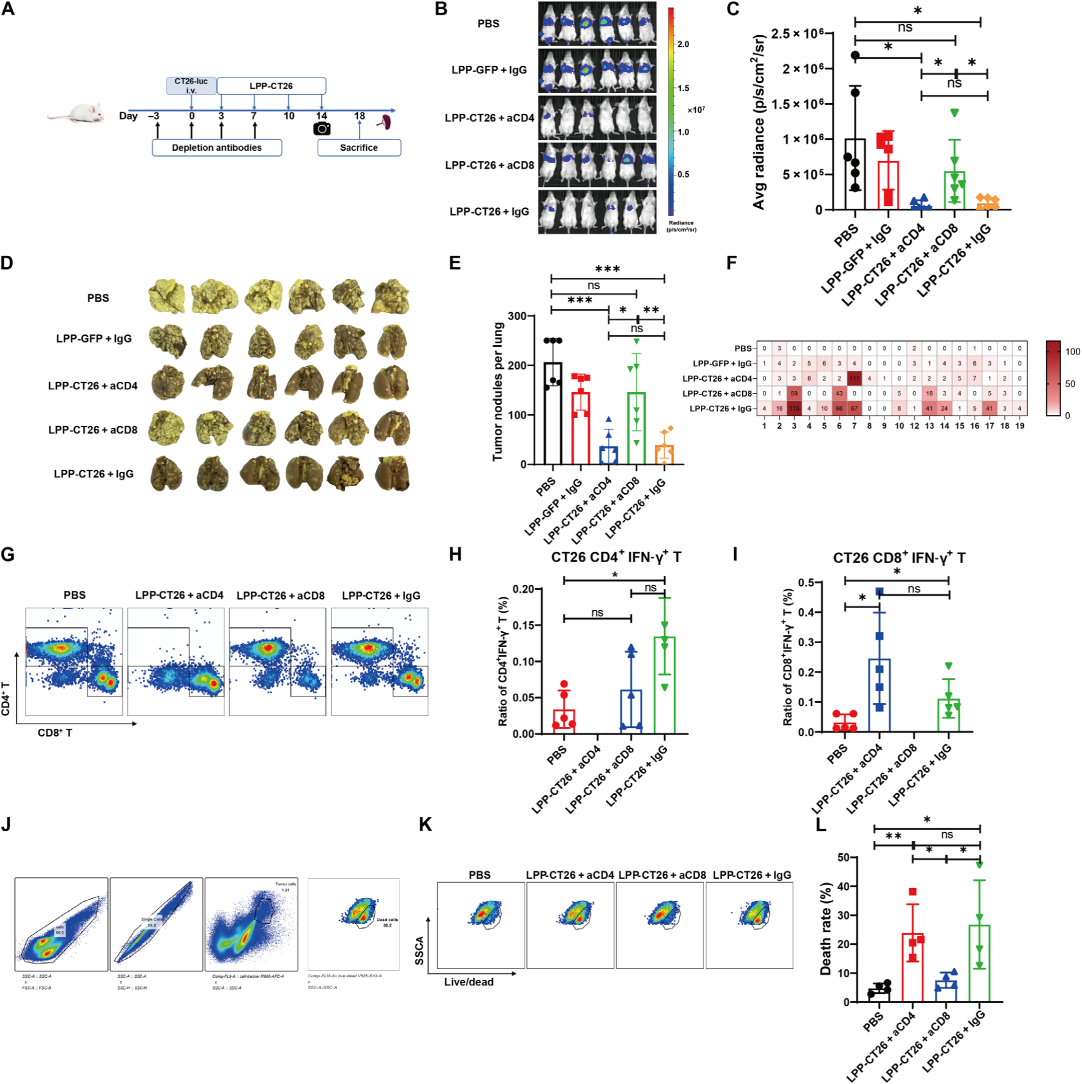
CD8^+^ T cell–mediated immune response effectively contributed to tumor control. (**A**) Experimental scheme and timeline for CD4^+^/CD8^+^ T cell depletion experiments. BALB/c mice (*n* = 6 per group) were administrated with T cell–depleting antibodies via intraperitoneal injection. CT26-luc cells were injected through tail vein on day 0 to establish the lung metastasis model. LPP-CT26 immunization was started from day 3. (**B**) In vivo imaging system (IVIS) images of CT26-luc tumors on day 14. (**C**) Fluorescence intensity of CT26-luc tumors in different groups on day 14. (**D**) Pictures of the lungs of mice at the time of euthanasia (day 18). (**E**) Quantification of the end point lung tumor nodules (day 18). (**F**) IFN-γ ELISpot assay of splenocytes from CT26-luc–bearing mice subjected to CD4^+^ or CD8^+^ T cell depletion. (**G**) FACS plots illustrating the effects of antibody-mediated depletion of CD4^+^ or CD8^+^ T cells in the mouse spleen. (**H** and **I**) ICS of IFN-γ by FACS in CD4^+^/CD8^+^ T cells in bulk splenocytes after T cell depletion. (**J**) FACS gating strategy for assessing tumor cell death after coculture of tumor cells with T cells. (**K**) Representative FACS images of CT26-luc cell death upon coculture with T cells in the presence of the indicated neutralizing antibodies. (**L**) Percentage of CT26-luc cells killed after coculture with T cells in vitro via FACS assay. Data were presented as means ± SD. One-way ANOVA statistical test was performed for all data analysis (*P < 0.05, **P < 0.01, ***P < 0.001).

To assess the relative significance of CD8^+^ versus CD4^+^ T cells in antitumor activities elicited by LPP-mRNA vaccines, in vivo anti-CD4 and anti-CD8 antibodies were used to deplete CD4^+^ and CD8^+^ T cells, respectively, before LPP-mRNA vaccine treatment in CT26-luc lung metastasis model (Fig. 5A) and MC38 subcutaneous xenograft model (fig. S11A). Notably, depletion of CD8^+^ T cells completely abolished the tumor-inhibitory effects of LPP-CT26, as supported by luciferase-based imaging analysis (Fig. 5, B and C). In contrast, depletion of CD4^+^ T cells showed minimal effects on LPP-CT26’s antitumor effects (Fig. 5, B to E). Similar results were observed in the MC38 model in that depletion of CD8^+^ but not CD4^+^ T cells abrogated the tumor-inhibitory potential of LPP-MC38 (fig. S11, B to E). IFN-γ ELISpot assays in splenocytes revealed that, while MHC-II neoepitopes were unable to stimulate IFN-γ production following the depletion of CD4^+^ T cells, MHC-I neoepitopes failed to promote the secretion of IFN-γ after the depletion of CD8^+^ T cells (Fig. 5F and fig. S11F). Depletion of CD4^+^ and CD8^+^ T cells following treatment with their respective antibodies was validated using flow cytometry (Fig. 5G and fig. S11G). We found, by ICS assays, that neither CD4^+^ nor CD8^+^ T cell depletion influenced the total IFN-γ secretion compared with the corresponding cancer vaccine immunization (Fig. 5, H and I, and fig. S11, H and I). We further investigated whether splenocytes from mice immunized with LPP-mRNA vaccine after depletion of T cell subsets could exert a killing effect on tumor cells in vitro (Fig. 5J and fig. S11J). As shown in Fig. 5 (K and L), splenocytes from CD8^+^ T cell–depleted mice showed little tumor cell killing. In contrast, those from CD4^+^ T cell–depleted mice exhibited robust tumor cell elimination much like the splenocytes from LPP-CT26–immunized mice that received immunoglobulin G injections. Comparable results were obtained in the MC38 syngeneic model (fig. S11, K and L). Our data, collectively, suggest that a CD8^+^ T cell–mediated immune response effectively contributes to tumor killing and growth control after LPP-mRNA vaccine immunization.

### Prophylactic immunization prevents tumor recurrence and induced memory T cells

**Fig. 6.**
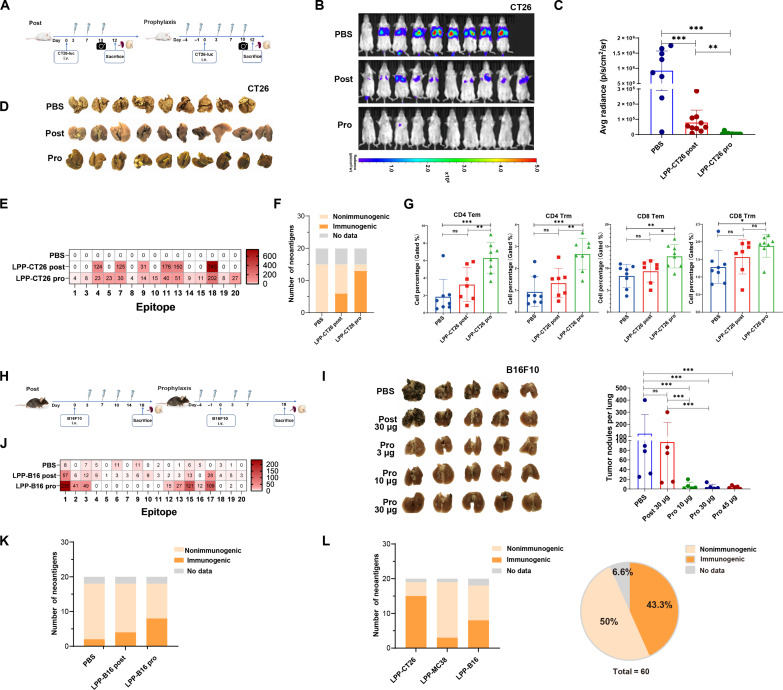
Prophylactic immunization with LPP-mRNA vaccine prevents lung colonization and induces memory T cells in CT26 and B16f10 syngeneic models. (**A** to **G**) Experiments in the LPP-CT26 model. Shown in (A) is the experimental scheme for the two different LPP-CT26 immunization schedules, i.e., prophylaxis (Pro) before intravenous injection of the CT26-luc cells and vaccination 3 days after CT26-luc cell injection (post). BALB/c mice (*n* = 9 or 10 per group) were immunized with 10 μg of LPP-CT26 in both settings. (B) to (E) show IVIS images (B) and fluorescence intensity of CT26-luc tumor burden in the lung (C) on day 10, the end point (day 12) lung images (D), and normalized IFN-γ ELISpot assay of splenocytes in CT26-luc lung metastasis mice (E). (F) Counts of neoantigen with and without immune response after LPP-CT26 prophylactic immunization versus post-immunization. (G). Percentage of CD4^+^/CD8^+^ T cell subsets including Tem and Trm cells in the spleen from mice treated with prophylactical versus post-administration. (**H** to **L**) Experiments in the B16F10 model. Shown in (H) is the treatment schedule for post versus prophylactic administration of the LPP-B16 vaccines in C57BL/6 mice bearing the B16F10 lung metastases (*n* = 10 per group). (I) Representative images of the end point lungs (left) and bar graphs of lung metastases (right) on day 18. (J) Normalized ex vivo IFN-γ ELISpot counts for the LPP-B16 neoantigens in splenocytes from indicated groups of mice (*n* = 5 per group). (K) Counts of neoantigens with or without immune response after LPP-B16 prophylactic versus post-immunization. (L) Summary of immunogenic versus nonimmunogenic neoantigens in the three animal models. Data were presented as means ± SD. One-way ANOVA test was performed for all data analysis (*P < 0.05, **P < 0.01, ***P < 0.001).

Prophylactic cancer vaccine immunization aims to simulate the preventive effects observed in post-surgical adjuvant therapy, potentially reducing the risk of recurrence in tumor-resected individuals. To test this, we used our “traditional” CT26-luc intravenous-injection model in which the mice received three LPP-CT26 treatments after CT26-luc tumor cell injections (Fig. 6A, left; Post), but in another cohort of mice, we also gave two additional doses of LPP-CT26 before CT26-luc cell injection, i.e., in a prophylactic (Pro) setting (Fig. 6A, right). As expected, the LPP-CT26 immunization after tumor cell injection greatly controlled lung tumor burden (Fig. 6, B to D). Two additional prophylactic LPP-CT26 vaccinations completely prevented lung colonization (Fig. 6, B to D). Furthermore, more neoantigens were recognized by T cells in the LPP-CT26 prophylactic group (13 of 15) compared to the LPP-CT26 post-administration group (6 of 15), as demonstrated by the IFN-γ ELISpot assays (Fig. 6, E and F). This heightened neoantigen-specific T cell response is indicative of the important role played by neoantigens in driving the prophylactic effect. In the event of antigen reexposure, such as when tumor cells are implanted, central memory T cells (Tcm) can rapidly differentiate into effector memory T cells (Tem) and tissue resident memory cells (Trm). We observed that prophylactic LPP-CT26 administration up-regulated both CD8^+^ and CD4^+^ Tem and Trm cells compared to post–LPP-CT26 treatment alone (Fig. 6G).

We also investigated the ability of the prophylactic LPP-mRNA vaccine to control tumor recurrence in the rapidly growing and immunologically cold B16F10 tumor model (Fig. 6, H to K). We introduced a fixed total dose of LPP-mRNA vaccine for both prophylactic and post-administration groups, with a total of four immunizations in each group (Fig. 6H). While post-tumor administration of LPP-B16 vaccine did not exhibit any noticeable effects on B16F10 lung colonization, prophylactic administration of LPP-B16, even at the lowest dose (10 μg), completely suppressed the outgrowth of intravenously injected B16F10 cells in the lung (Fig. 6I). IFN-γ ELISpot assays in splenocytes further demonstrated that prophylactically deployed LPP-B16 vaccine elicited more robust neoepitope-specific responses compared to post-tumor vaccination (Fig. 6J), and prophylactic administration at the same dose (30 μg) also elicited a more robust and broader specific T cell response (Fig. 6K). These findings collectively highlighted the potential of prophylactic mRNA vaccine as an adjuvant therapy in the prevention of tumor recurrence.

Preceding studies unveiled that the LPP-mRNA vaccines elicited antitumor activities and favorable immune responses in all three murine tumor models (i.e., CT26, MC38, and B16F10). Among the total pool of 60 neoantigens, a noteworthy 26 neoantigens incited an immune response (Fig. 6L). This positive (immunogenic) neoantigen prediction rate of 43.3% (Fig. 6L) surpasses those reported in clinical studies thus far (*15*, *34*), and highlights the precision of our unique algorithms and accentuates the promising potential of neoantigen-based vaccination strategies in effectively inducing neoantigen-specific immune responses.

### LPP mRNA vaccine protects mice from tumor rechallenge by evoking memory T cell response

**Fig. 7.**
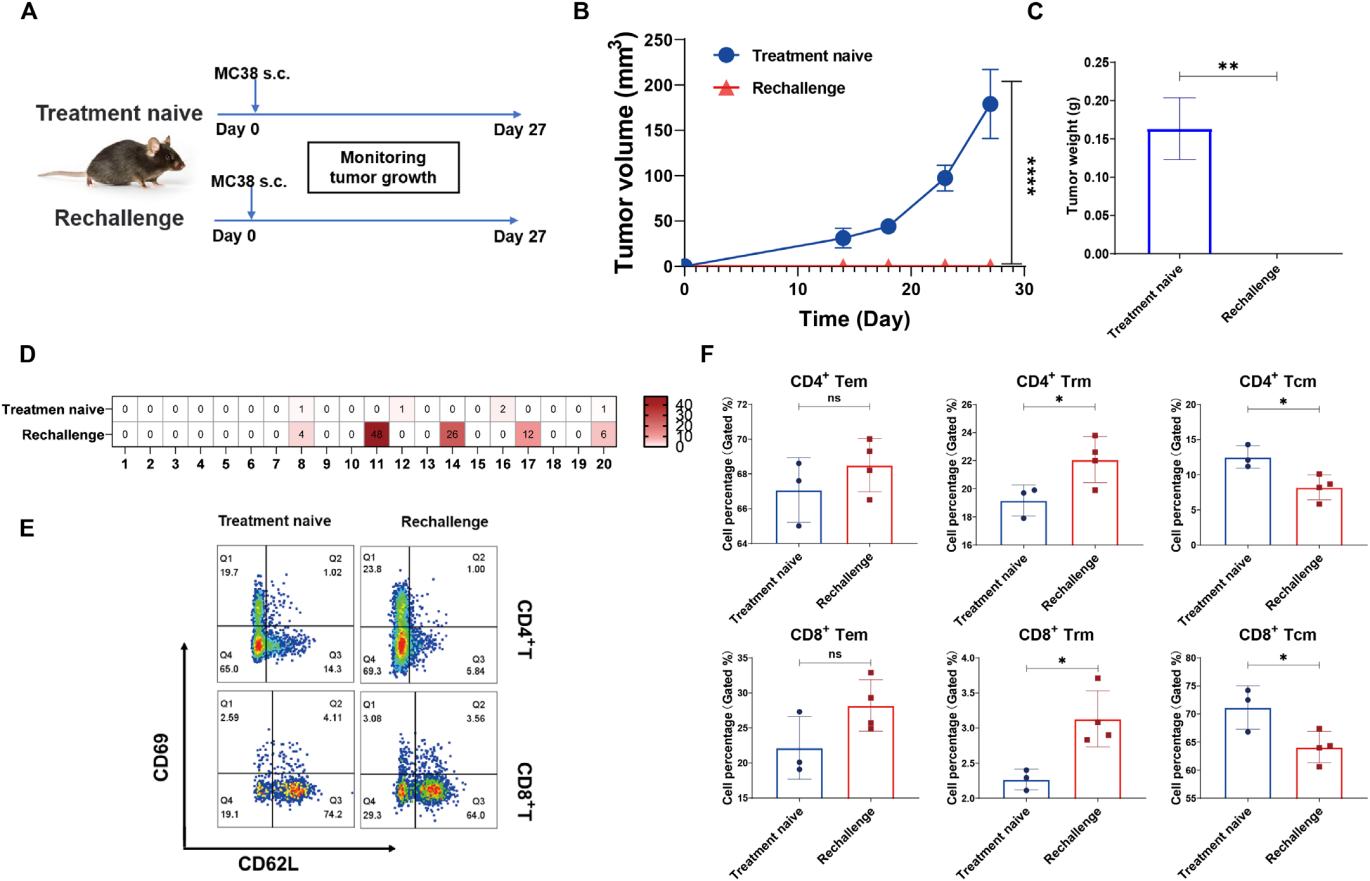
LPP-mRNA vaccine immunization establishes protective T cell memory and protects mice from tumor cell rechallenge. (**A**) Experimental scheme. Both treatment-naïve and MC38 tumor–regressing mice were inoculated with MC38 cells subcutaneously. Tumor growth was monitored, and mice were euthanized on day 24. (**B** and **C**) Tumor growth curves until day 27 (B) and the end point tumor weight (C). (**D**) Individual peptide restimulation for IFN-γ ELISpot assay in bulk splenocytes. (**E** and **F**) Representative Tcm, Tem, and Trm in the splenocytes as assayed by flow cytometry (E) and quantitative summary (F) of Tcm, Tem, and Trm in T cell subtypes in the splenocytes from the treatment-naïve and rechallenged mice. Data were presented as means ± SD. One-way ANOVA statistical test was performed for all data analysis (*P < 0.05, **P < 0.01).

Next, we evaluated the effects of LPP-mRNA vaccine immunization on tumor immune memory and the long-term impact on tumor control using the MC38 model. We showed earlier that MC38-bearing mice immunized with LPP-MC38 had much longer survival with regressing tumors and without tumor recurrence during the 6-month follow-up (Fig. 4, A to D). Herein, we took a group of such preimmunized mice with regressing MC38 tumors and paired with a group of age-matched treatment-naïve mice and then implanted identical numbers of MC38 tumor cells in the left flank of both groups of mice (Fig. 7A). While all treatment-naïve mice exhibited robust tumor growth with large end point tumors, the MC38-rechallenged mice remained tumor-free throughout the experimental duration (Fig. 7, B and C), suggesting functional memory T cell response against MC38 cell neoantigens. Compared to splenocytes from treatment-naïve mice, the splenocytes from the rechallenged mice displayed a stronger response to the original LPP-MC38 neoepitopes as revealed by the IFN-γ ELISpot assays (Fig. 7D). Investigating the profile of memory T cells, we observed a notable reduction in Tcm but a substantial increase in both Tem and Trm subpopulations of the CD4^+^ and CD8^+^ T cells (Fig. 7, E and F). These results suggest that upon receiving antigen restimulation (i.e., MC38 tumor rechallenge), Tcm likely differentiated into Tem and Trm where Tem secreted effectors such as IFN-γ and IL-4 for protective immune memory, while Trm emerged as a pivotal part of TILs staging immune surveillance and “safeguarding” the tumor microenvironment. In summary, our findings (Fig. 7) strongly suggested that the LPP-mRNA vaccine induced a robust and functional memory T cell response capable of rapidly executing cytotoxic antitumor activities upon reexposure to the antigen.

### a-PD1 combination therapy prolongs the survival of tumor-bearing mice

**Fig. 8.**
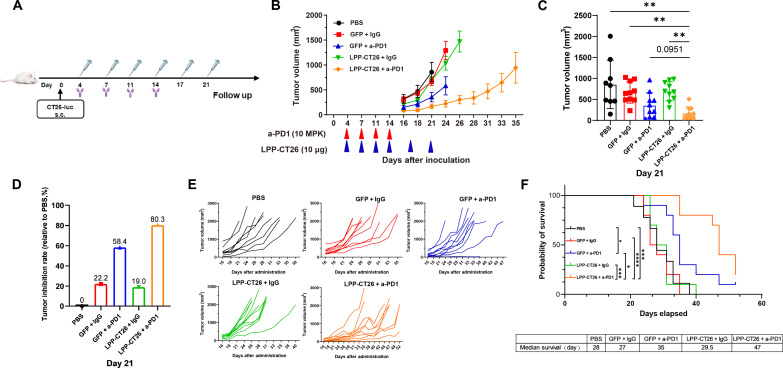
LPP-mRNA vaccine combined with ICI prolongs the survival of mice bearing a-PD1 refractory tumors. (**A**) Experimental scheme. BABL/c mice were subcutaneously implanted with CT26-luc tumor cells and then immunized with LPP-CT26 (or control LPP-GFP) six times twice a week at 10 μg dosage and with four intraperitoneal doses of a-PD1 or control immunoglobulin G (*n* = 10 per group). Tumor volume and animal survival were monitored every 3 days. (**B**) Tumor growth curves. MPK, mg per kilogram (body weight). (**C**) Bar graph presentation of tumor volumes measured on day 21. (**D**) Tumor inhibition rate of each treated group compared to the control group. (**E**) Growth curves of individual CT26 tumors. (**F**) Survival rate in mice bearing the subcutaneous CT-26 tumors treated with LPP-CT26 and/or a-PD1. The end point for animal survival was marked as the tumor reaching 2000 mm^3^. Data were presented as means ± SD. One-way ANOVA statistical test was performed for all data analysis *T* test (*P < 0.05, **P < 0.01, ***P < 0.001, ****P < 0.0001).

CT26 tumor is known to be resistant to programmed death-1/programmed cell death-ligand 1(PD1/PDL1) ICI therapy (*35*). In light of relatively limited tumor suppression on CT26 tumors even with a high dose (30 μg) of LPP-CT26 immunization (Fig. 4G), we investigated the potential synergistic effects of LPP-CT26 with an anti-PD1 (a-PD1) ICI (Fig. 8). The underlying hypothesis posited that a-PD1 monotherapy could improve the tumor microenvironment by promoting an inflamed state that is nonexcluded and less suppressive, thereby facilitating infiltration of neoantigen-specific T cells into tumors. This, in turn, was expected to augment the antitumor efficacy of the LPP-mRNA vaccine. Briefly, we combined a suboptimal dose of LPP-CT26 (10 μg) and a-PD1 in the subcutaneous CT26 tumor model (Fig. 8A) and found that the combination therapy markedly retarded tumor growth compared to a-PD1 or LPP-CT26 monotherapies (Fig. 8, B and E) leading to smaller end point tumor volume (Fig. 8C), higher tumor inhibition rate (Fig. 8D), and more prolonged animal survival (Fig. 8F). The tumor inhibition rate in the combination group reached 80.3%, much higher than 19.0% for LPP-CT26 and 58.4% for a-PD1 monotherapy (Fig. 8D). Furthermore, survival (evaluated as the time for the tumor to reach 2000 mm^3^) analysis indicated that the median survival for the combination was 47 days, much longer than the 29.5 days for LPP-CT26 (*P* < 0.001) and 35 days for a-PD1 (*P* < 0.05) (Fig. 8F).

Given the reliance of LPP-PCV’s efficacy on successful neoantigen-specific T cell infiltration, adding a-PD1 to the vaccine platform appears to effectively impede tumor growth in a-PD1 refractory tumors. This combination strategy is currently being evaluated in various clinical trials, showing promising clinical outcomes for advancing vaccine development in the realm of cancer treatment (*36*, *37*).

### In vivo administration of LPP-mRNA vaccine is safe

**Fig. 9.**
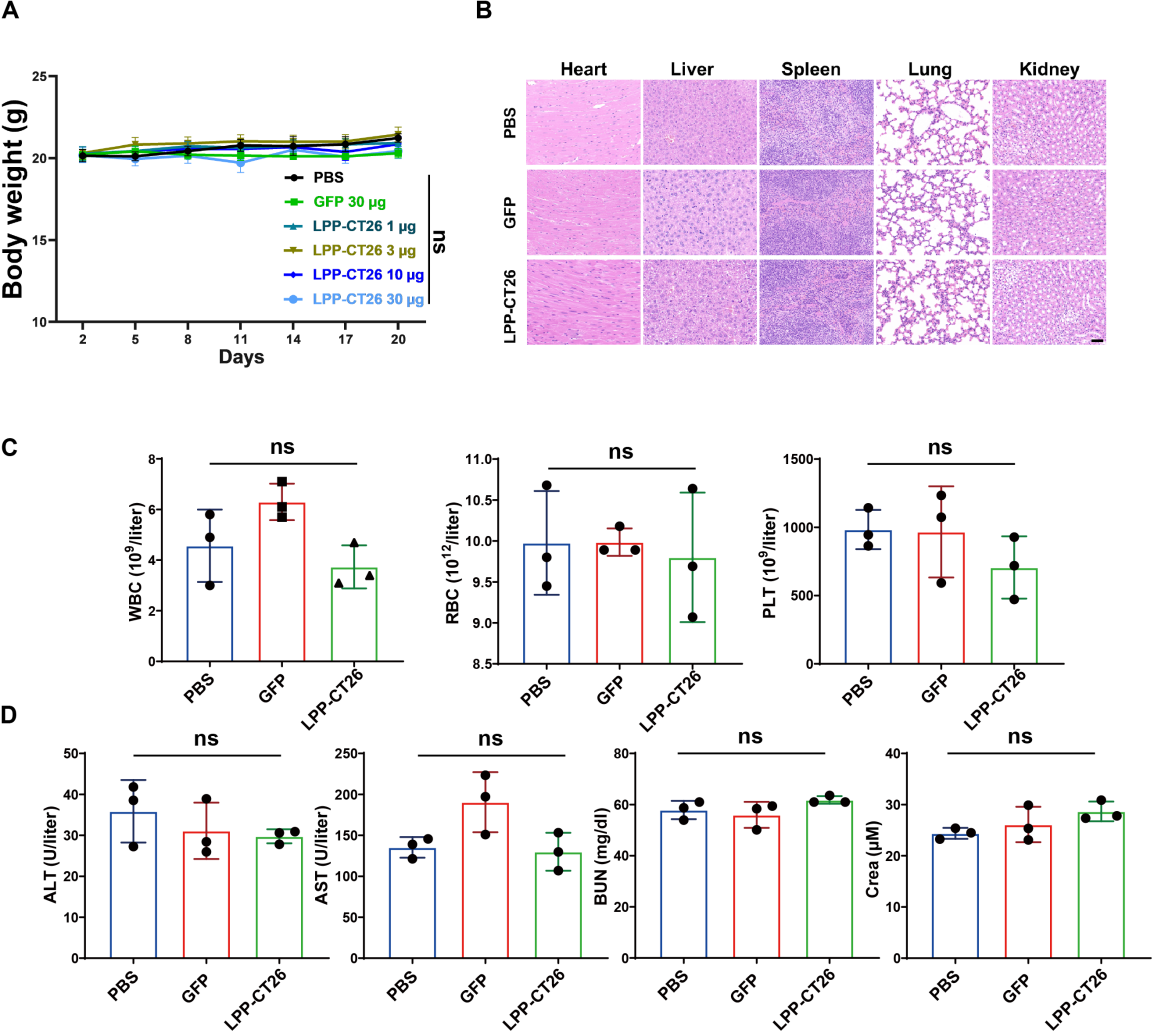
Safety evaluation of LPP-mRNA vaccine in vivo. (**A**) Body weight of MC38-bearing mice during the LPP-mRNA vaccine immunization. (**B**) Representative hematoxylin and eosin images of heart, liver, spleen, lung, and kidney in different groups. Scale bar, 20 μm. (**C**) Representative blood cell counts in mice after immunization with LPP-CT26 or LPP-GFP vaccine. WBC, white blood cells; RBC, red blood cells; PLT, platelets. (**D**) Representative blood biochemical parameters in mice after immunization with LPP-MC38 or LPP-GFP vaccine. ALT, alanine transaminase; AST, aspartate transaminase; BUN, blood urea nitrogen; Crea, creatinine. Data were presented as means ± SD. One-way ANOVA statistical test was performed for all data analysis.

To determine the in vivo safety of the LPP-mRNA vaccine, we monitored the body weight of mice and did not observe any distinct decrease in body weight of experimental mice, even at high doses of LPP-mRNA vaccine immunization (Fig. 9A). In addition, hematoxylin and eosin staining of major organs did not reveal discernible pathologies or inflammatory lesions in mice that received LPP-mRNA vaccines (Fig. 9B). Furthermore, the tumor-bearing mice immunized with our LPP-mRNA vaccines showed no notable abnormalities, both quantitatively and qualitatively, in white blood cells, red blood cells, and platelets (PLT) (Fig. 9C). Last, the blood biochemistry, including alanine aminotransferase (ALT), aspartate aminotransferase (AST), blood urea nitrogen (BUN), and creatinine (Crea), did not differ between vaccinated and control mice (Fig. 9D), suggesting normal liver and kidney functions. These results, collectively, suggest that the administration of our LPP-mRNA vaccines is safe in vivo.

### LPP-PCV vaccination elicits neoantigen-specific T cell response in patients

**Fig. 10.**
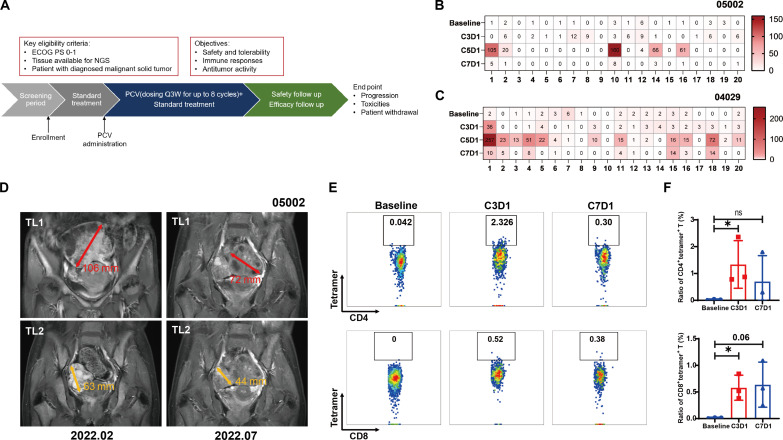
LPP-PCV vaccination elicits neoantigen-specific T cell response in patients. (**A**) Diagram of the clinical design for the LPP-PCV treatment. (**B** and **C**) IFN-γ ELISpot assay of PBMCs after different cycles of LPP-PCV immunization from patient 05002 (B) and patient 04029 (C). (**D**) Representative MRI images of two major target lesions (TL1 was highlighted in red, and TL2 was highlighted in yellow) in patient 05002. Note that 5 months after vaccination, the diameters of both TLs were reduced. (**E** and **F**) FACS plots (E) and bar graphs (F) showing the LPP-PCV–induced increases in patient 04029 PBMCs of both CD4^+^ and CD8^+^ T cells positive for de novo–primed neoantigen #1 tetramers. Data were presented as means ± SD. One-way ANOVA statistical test was performed for all data analysis (*P < 0.05).

The favorable preclinical outcomes observed in safety and efficacy studies using a clinical-grade LPP-mRNA vaccine in murine models have propelled the momentum toward clinical translation. In this context, a phase 1 clinical trial (NCT05198752) involving our LPP-PCV, denoted as SW1115C3, has been initiated for patients with advanced solid tumors in Australia since January 2022. Following our preclinical findings, another investigator-initiated trial at Shanghai East Hospital (www.chictr.org.cn; identifier ChiCTR2100050688) to evaluate the safety and efficacy of our LPP-mRNA PCV was carried out in patients with malignant tumors (Fig. 10A). Following the screening, we included two subjects (Fig. 10): one with advanced gastric cancer with multiple ovary metastases and many lines of prior treatment (patient id 05002), and the other with early-stage breast cancer after radical mastectomy (patient id 04029). Patient 05002, a 52-year-old woman diagnosed with advanced gastric cancer with ovarian implantation metastases, underwent multiple prior treatments, including EOX (epirubicin, oxaliplatin, and capecitabine) chemotherapy, sintilimab (an a-PD1 antibody), albumin paclitaxel, FOLFIRI (fluorouracil, folinic acid, and irinotecan) chemotherapy, and Tegio (a fluorouracil derivative). Because of the substantial tumor burden and advanced tumor stage, the patient failed all preceding regimens and ended with rapid disease progression. The anticipated overall survival for individuals in such conditions ranged from only 3 to 6 months. However, with the combined administration of LPP-PCV, vedolizumab (a humanized anti-human epidermal growth factor receptor 2 antibody conjugated with monomethyl auristatin E) and pembrolizumab since 03 February 2022, this patient achieved a progression-free survival of 8.4 months. IFN-γ ELISpot assays of the patient’s peripheral blood mononuclear cells (PBMCs) detected neoantigen-specific T cell responses after four cycles (i.e., during C5D1) of vaccination (Fig. 10B). During vaccination, the patient experienced only grade 1-2 adverse events of low-grade fever and redness and swelling at the injection site. Periodic radiographic imaging showed a substantial reduction in tumor size at the 6th month (target lesion maximal diameter of 107 mm) compared to baseline (target lesion maximal diameter of 178 mm) (Fig. 10D). The most favorable treatment evaluation during the LPP-PCV combination therapy was partial remission, marked by a substantial reduction in the patient’s ovarian metastatic lesions (Fig. 10D).

The other participant, patient 04029, diagnosed with luminal B breast cancer, underwent complete tumor tissue removal following neoadjuvant therapy. After surgery, she received LPP-PCV in combination with letrozole and fulvestrant endocrine therapy. During the vaccination period, no grade 3-4 severe adverse events occurred, and this patient experienced only low-grade fever and redness, swelling, and hard nodules at the injection site, all of which dissipated within 3 days after vaccination. After six cycles of LPP-PCV immunization, we observed a robust specific T cell response to 11 of the 20 neoantigens (Fig. 10C and fig. S12). One year postoperatively, follow-up tumor imaging did not reveal any signs of tumor recurrence or metastasis, and continuous tumor monitoring has been ongoing. The p-MHC tetramer of neoantigen #1 was constructed, and neoantigen-specific T cells could be detected by flow cytometry in the peripheral blood of patient 04029 after two and six cycles of LPP-PCV vaccination (Fig. 10, E and F, and fig. S13). Unfortunately, because of the paucity of PBMCs collected after the patient’s four-cycle LPP-PCV vaccination, relevant flow cytometry data for that time point were unavailable.

In these two patients, we found that in the settings of both palliative and postoperative adjuvant treatment of patients with tumors, the administration of LPP-PCV on top of the existing treatment could stimulate neoantigen-specific T cells and elicit meaningful antitumor effects. Nevertheless, rigorous randomized controlled trials are needed to determine the clinical efficacy and potential survival benefits of LPP-PCV in patients with cancer.

## DISCUSSION

In this study, we introduced an innovative personalized mRNA cancer vaccine encoding a set of 20 in silico predicted neoantigens, leveraging the distinctive core-shell architecture of the proprietary LPP mRNA delivery system (*25*, *28*). The therapeutic efficacy of the LPP-mRNA cancer vaccine was demonstrated in three murine tumor models: CT26, MC38, and B16F10. Moreover, we explored the optimal administration protocol for delivering the LPP-mRNA vaccines to attain an enhanced antitumor response. Mechanistically, the vaccination led to the activation of neoantigen-specific cytotoxic T cells, triggering the secretion of cytotoxic cytokines. Through antibody-mediated cell depletion, we subsequently showed that the LPP-mRNA vaccine was mainly dependent on CD8^+^ T cells for its killing effect and antitumor activities. The LPP-mRNA vaccines also established enduring T cell memory, endowing protection against subsequent tumor cell rechallenge. Promisingly, after LPP-PCV vaccination, two patients manifested neoantigen-specific T cells and showed potential clinical benefits (with reduced target lesions) without serious adverse events. Thus, our study not only pioneered an mRNA cancer vaccine platform but also offered its potential underlying mechanisms of action.

The most critical initial step in cancer vaccine development is the screening and identification of tumor-specific neoantigens. The establishment of high-quality and ultimately immunogenic neoantigens necessitates a comprehensive assessment of multiple vital factors encompassing, among others, frequency of DNA mutations within the somatic cells of tumor tissue, expression levels of genes and transcripts, affinity of peptides to MHC molecules, the stability of peptide-MHC binding, the potential for peptide translocation to the cell surface, and the compatibility of the peptide with T cell receptor recognition (*38*, *39*). Our delivery platform incorporated a liner mRNA containing 20 neoantigens of 27-mer in LPP. Notably, our precision of neoantigen identification as facilitated by SmartNeo surpasses the 43% threshold in our investigative work, a performance metric that outperforms other public neoantigen screening algorithms (*15*, *23*). The LPP-mRNA vaccines we designed contained MHC-I and MHC-II neoantigens with the potential to prime both CD4^+^ and CD8^+^ T cells. However, cell depletion experiments showed that the ability of the LPP-mRNA vaccine to inhibit tumor growth was greatly weakened after depletion of CD8^+^ T cells, thus causatively implicating the CD8^+^ T cells in mediating its tumor-suppressive effects. Although the importance of CD4^+^ T cells and MHC class II–restricted neoepitopes in cancer vaccine efficacy has been previously reported (*40*, *41*), our current findings do not support a major role for them in mediating the antitumor effects of the LPP-mRNA vaccines. More in-depth exploration of their involvement and underlying mechanisms is warranted.

Clinical trials often lack a standardized administration regimen closely tailored to specific clinical indications and vaccine formulations. For instance, DNA-based cancer vaccines often require complex delivery methods like electroporation or jet injection, which poses practical challenges for clinical implementation (*42*, *43*). In contrast, mRNA cancer vaccines could be administered by simple subcutaneous or intramuscular injection to provide sufficient neoantigen delivery to APCs (*34*, *44*). A prior study demonstrated the benefits of distributing the vaccine doses across multiple draining lymphatic regions to enhance antigen-specific T cell responses (*45*). Our study also observed that modifying the approach to subcutaneous injection and multisite administration led to rapid and enhanced immune response and antitumor efficacy. This observation could offer valuable insights into antigen kinetics, which might facilitate further refinement of clinical trials through exploration in murine models. Of significance, we expanded the therapeutic scope of our LPP-mRNA vaccine platform from treatment to prophylactic settings, establishing a conceptual framework for its potential in preemptive prevention of tumor recurrence. The strategic administration allows priming of tumor-specific T cells in mice before tumor cell implantation. This tactical approach equips mice with the ability to swiftly activate Tem cells upon reexposure to the corresponding tumor neoantigens, promptly eradicating incipient microscopic metastatic lesions within the body, as demonstrated in our study (*23*). These findings not only hold implications for the future application of LPP-PCV in postoperative adjuvant cancer therapy but also offer a valuable paradigm for harnessing the prophylactic potential of this approach in averting tumor recurrence.

Our work further suggests that the combination of LPP-mRNA vaccines with ICIs holds the potential to enhance responses in a-PD1–resistant tumors, ultimately extending the survival of mice. Our preclinical results are consistent with emerging clinical studies, particularly in patients with melanoma, where personalized neoantigen cancer vaccines have demonstrated clinical feasibility and safety. However, prior clinical results of ICIs combined with a PCV showed similar overall response rates, progression-free survival, and overall survival compared to ICI treatment alone (*46*, *47*). Whether personalized neoantigen vaccines in combination with a-PD1 may play a role in the therapy of patients with cancer should be considered carefully. Excitingly, a phase 2b clinical trial (KEYNOTE-942) has yielded remarkable success, which investigated the efficacy of their PCV mRNA-4157 in combination with pembrolizumab for patients with stage III/IV melanoma who had undergone complete resection. Noteworthy achievements included the attainment of the primary end point, with the combination treatment leading to a substantial reduction in the risks of recurrence and mortality [hazard ratio = 0.56 (95% CI, 0.31 to 1.08), *P* = 0.0266], (*36*). It can be envisioned that, in the foreseeable future, the synergistic utilization of ICIs and PCVs, with judicious patient selection, could considerably enhance clinical outcomes. Another pivotal revelation from our study is establishing enduring and robust T cell memory through LPP-PCV vaccination. It appears that subsequent to the apparent eradication of tumors, the neoantigen-specific memory T cells exhibited the ability to orchestrate potent immune surveillance and swiftly eliminate rechallenged target tumor cells (*48*).

Despite the exciting advances our current study brings to the field, we are cognizant of several limitations and caveats associated with the LPP-mRNA vaccine platform. First, the prevalent clonal and tumor cell heterogeneity that preexists in treatment-naïve tumors, coupled with in vivo immune editing, may give rise to nonresponsive tumor variants adept at evading immune recognition, thus obviating the antitumor effects of the LPP-mRNA vaccines. Second, our study primarily used murine tumor models, which inherently differ from the human immune system, precluding a complete replication of immune responses in cancer patients. Unfortunately, current humanized mouse models fall short in fully reconstituting myeloid cells and achieving the delivery of human-derived cancer neoantigens, and obtaining PBMCs corresponding to HLA typing for cell line– or patient-derived xenografts presents challenges, thus hindering the establishment of a suitable humanized immune system in mice for preclinical exploration. Third, the choice of indications may be crucial in applying LPP-PCV. Vaccine efficacy requires an optimally functioning host immune system; therefore, optimal vaccine efficacy hinges on a host’s immune system operating at its peak, implying that LPP-PCV may exert greater effectiveness within a postoperative adjuvant setting as opposed to palliative care for patients with heavily pretreated advanced cancers. Last, to substantiate its antitumor efficacy and potential clinical utility, the LPP-PCV needs to be deployed in more prospective clinical trials to accumulate additional patient data.

In summary, we have developed an LPP-formulated mRNA cancer vaccine that can elicit robust neoantigen-specific T cell responses, induce durable immune memory, and effectively control tumor growth in multiple syngeneic murine tumor models. In addition, a combination of vaccine immunization and ICI exhibits prominent antitumor activity in ICI-refractory tumors. The study lays a solid foundation for future clinical investigation of LPP-based mRNA vaccines.

## MATERIALS AND METHODS

### Cancer cell lines and WES and RNA sequencing

Cancer cell lines (B16F10, CT26, and MC38) were routinely cultured. A total of 1 × 10^6^ of each cell line were harvested and for subsequent WES and RNA sequencing (RNA-seq, Novogene). WES assay is based on the Illumina Novaseq 6000 sequencing technology platform, and then the kit was used to construct libraries and obtain sequencing data from samples of acceptable quality. RNA-seq assay is based on the Illumina Novaseq 6000 sequencing technology platform to perform transcriptome sequencing and obtain sequencing data of qualified samples. The data were released to further neoantigen prediction.

### Neoantigen prediction and sequence design

The patent for the SmartNEO algorithm has been filed by StemiRNA Therapeutics Inc., and details will become publicly available when patent application is approved. For murine neoantigen prediction, the WES and RNA-seq data of murine tumor cells were matched to the gene database of the same strain of mice to obtain mutation information. For neoantigen prediction in patients, tumor tissue and PBMCs of enrolled patients were obtained, and WES was performed on tumor tissue and PBMCs, respectively, while bulk RNA-seq was performed on tumor tissue to obtain the corresponding transcriptomic data. WES data from tumor tissue, WES data from PBMCs, and RNA-seq data from tumor tissue were mapped to the human reference genome to obtain their respective sequence comparison results. The subsequent prediction process was similar between mice and patients, including determining the haplotype typing of DNA variants in normal control germ cells, DNA variants in tumor tissue somatic cells, and the ensemble of DNA variants in both (based on the results of sequence matching) and determining the mutation frequency of DNA variants in tumor tissue somatic cells at the level of RNA data based on the results of sequence matching of tumor tissue RNA and the results of DNA variants in tumor tissue somatic cells. On the basis of the results of sequence comparison of tumor tissue RNA, the number of sequences corresponding to each transcript was determined, and the number of sequences of different transcripts from the same gene was summed up, thus determining the expression levels of transcripts and genes. The results of haplotype typing were annotated for DNA variants in somatic cells of tumor tissues, normal control germ cells, and somatic cells of tumor tissues for the ensemble of both DNA variants. MHC typing was determined on the basis of the results of tumor tissue DNA sequence alignment. Candidate neoantigen polypeptide sequences based on the annotated DNA variation results of tumor tissue somatic cells, the haplotype typing results of the annotated DNA variation ensemble of both normal control germ cells and tumor tissue somatic cells, and the MHC typing results were subsequently obtained. High-quality neoantigens were screened and identified on the basis of the combined scoring of the aforementioned characteristics of the candidate neoantigens, including mutation frequency of DNA in tumor tissue somatic cells, mutation frequency of DNA in normal control germ cells, mutation frequency of tumor tissue somatic cell DNA variants at the level of RNA data, the expression level of genes and transcripts, the mutant peptide–MHC affinity, the WT peptide–MHC affinity, and the mutant peptide–MHC affinity. Peptide-MHC affinity ratio, mutant peptide–MHC binding stability, immunogenicity of the mutant peptide, and the candidate neoantigens were ranked according to a composite score.

High-quality tumor neoantigen sequences were selected for mRNA template construction based on the sequencing results. The amino acid sequence structure of the open reading frame region was as follows: SP-Linker-neoantigen1-linker-neoantigen2-…-linker-MITD. The start linker sequence is GGSGGGGSGG, the middle linker sequence is GGSGGGGSGG, and the end linker sequence is GGSLGGGGSG. The signal peptide (SP) sequence is MRVTAPRTLILLLSGALALTETWAGS, and the MITD sequence is VGIVAGLAVLAVVVIGAVVATVMCRRKSSGGKGGSYSQAASSDSAQGSDVSLTA. The 5′UTR(5′-3′) sequence is **AG**GAAAUUCCAUUUGGCUGCAGCUUCUGGAGGGAGCCGACAGGAGACGUGGGGAGACG**GCCACC**. The beginning “AG” is required for capping during transcription using the GAG trimer cap, and the underlined sequence is the Kozak sequence. The 3′UTR(5′-3′)sequence is GCUGCCUUCUGCGGGGCUUGCCUUCUGGCCAUGCCCUUCUUCUCUCCCUUGCACCUGUACCUCUUGGUCUUUGAAUAAAGCCUGAGUAGGAAGU. The 3′UTR sequence is derived from the 3′UTR region of the α-globin gene. The PolyA tail length is 75 nt. We used N1-methylpseudouridine-5-triphosphate for nucleoside modification to synthesize less immunogenic and more stable mRNAs.

The coding sequences for each of the individual neoantigens are concatenated to form a single, more extended sequence representing the entire set of tandem neoantigens. LinearDesign is then used to optimize the stability and codon usage for this concatenated sequence as a whole (*24*). The algorithm aims to find the mRNA sequence with the lowest minimum free energy among all possible sequences encoding a specific protein. LinearDesign also optimizes codon usage by incorporating the codon adaptation index (CAI). CAI measures how closely the codon usage in a sequence matches the optimal codon usage for a particular organism, which can enhance translation efficiency.

### mRNA vaccine particle preparation

Plasmids containing individual-specific neoantigen sequence designs were obtained by Gibson Assembly, and the target plasmids were transformed into receptor cells to prepare glycerobacteria. The obtained bacteria were subjected to plasmid preparation using the NucleoBond PC 10000 EF kit extraction process. After preparing the plasmid DNA template, DNA template purification, in vitro transcription, DNA template removal, mRNA purification, and filtration, we obtained a stable mRNA stock solution. LPP nanoparticles were prepared using a two-step method. Briefly, protamine sulfate was dissolved in 25 mM sodium acetate (pH 5.2) and diluted with ribonuclease-free water. mRNA/protamine complexes were prepared by mixing the mRNA solution (pH 4, 10 mM citrate buffer) and protamine sulfate solution at a volume ratio 5:1 and standing for 30 min at room temperature. After that, ionizable lipid, DOPE (Avanti Polar Lipids, Birmingham, USA), cholesterol (A.V.T. Pharmaceutical, China) and mPEG-DMG (Avanti Polar Lipids, Birmingham, USA) were dissolved in ethanol at molar ratio of 40:15:43.5:1.5. The N:P ratio is 4.8:1. The lipid mixture was combined with mRNA/protamine complexes at a volume ratio of 3:1 (aqueous:ethanol) using a microfluidic mixer (Inano D, Micro&Nano Technology Inc., China). Formulations were dialyzed against PBS (pH 7.4) in dialysis cassettes for at least 12 hours. Formulations were concentrated using Amicon ultracentrifugal filters (EMD Millipore, USA), passed through a 0.22-μm filter, and stored at 4°C (*49*).

### Synthetic peptides

We ordered the peptide containing the neoantigen mRNA encoding AA from GenScript (Jiangsu, China) with a peptide length of 27-mer. The lyophilized peptides were dissolved in dimethyl sulfoxide (Sigma-Aldrich, D2650) for use, and the working concentration for the assay was 2 μg/ml. Specific peptide sequence information is provided in table S1.

### Characterization of nanoparticles

The size distribution and zeta potential of the LPP-mRNA vaccine were measured with dynamic light scattering Zetasizer (Zetasizer Nano, Malvern Pananalytical Inc., Westborough, MA, USA). The morphology of the LPP-mRNA vaccine was detected by TEM (TEOL JEM-2100 transmission electron microscope).

### Cell lines and cell culture

The 293T cells were obtained from the National Collection of Authenticated Cell Cultures (Shanghai, China) and were cultured in RPMI-1640 containing 10% fetal bovine serum (FBS) and 1% penicillin-streptomycin (PS). The murine cells B16F10, CT26-luc, and MC38 were also acquired from the National Collection of Authenticated Cell Cultures (Shanghai, China) and were cultured in Dulbecco’s modified Eagle’s medium (DMEM)/RPMI-1640 containing 10% FBS and 1% PS. The cell culture was maintained at 37°C with 5% CO_2_ in the incubator.

### Cellular uptake

For the cellular uptake assay, 293T and CT26-luc cells were seeded into six-well culture plates 5 × 10^5^ per well and cultured for 24 hours. Then, different concentrations (0.0625, 0.125, 0.25, 0.5, 1, 2, and 4 μg/ml) of LPP-GFP were added into wells at 37°C. Twenty-four hours later, the cellular uptake assay was carried out using fluorescence microscope and flow cytometry, according to the different concentrations.

### Cytotoxicity assays

The 293T cells were seeded in 96-well plates and cultured for 24 hours. The different concentrations of LPP-CT26 were added to the plates and cocultured for 24 hours. After the coculture for 24 hours, 100 μl of RPMI-1640 medium containing 10 μl of CCK-8 solution was added to each well and incubated for another 2 hours. The absorbance of the CCK-8 solution was measured at 450 nm by a microplate reader.

### Flow cytometry analysis (FACS)

To detect different types of cells by fluorescence-activated cell sorting (FACS), tumor cells, splenocytes, and cells in the lymph nodes were harvested. Monoclonal antibodies for extracellular staining included Live/Dead (Thermo Fisher Scientific, L34957), CD3 (BioLegend; 100210, 100203, 100206, and 344818), CD4 (BioLegend; 100424, 130308, and 300532), CD8a (BioLegend; 100734, 100722, 162306, and 344710), CD11b (BioLegend, 101235), CD11c (BioLegend, 117324), CD44 (BioLegend, 103039), CD62L (BioLegend, 161204), CD69 (BioLegend, 164204), CD86 (BioLegend, 159220), CD103 (BioLegend, 121421), and I-A/I-E (MHCII) (BioLegend, 107608). ICS was performed with the antibody against IFN-γ (BioLegend, 506524), TNF-α (BioLegend, 506327), IL-2 (BioLegend, 503822), and IL-4 (BioLegend, 144806) using the cytofix/cytoperm kit (BD Pharmingen) after stimulation of 2 × 10^6^ splenocytes with neoantigen peptides (2 μg/ml) in the presence of brefeldin A (20 μg/ml; BioLegend, 420601) and protein transport inhibitor (40 μg/ml; BD Pharmingen, 554724) for 18 hours at 37°C. FACS was performed to detect the expression of intracellular cytokines. To detect neoantigen specific T cells, the cells were stained with T cell–specific p-MHC (Haoshengmed, Shanghai, China) following the manufacturer’s instructions. When the cells were ready, they were collected for further detection by BD LSR II flow cytometer (BD Biosciences) and analyzed. All flow cytometry results were analyzed with the FlowJo v10 software.

### Tissue distribution studies

ICR mice were inoculated with LPP containing GFP mRNA at 2, 6, 12, 24, 48, 72, and 168 hours. After the mice were euthanized, the mRNA content of GFP in each organ of mice was measured by RT-PCR after RNA extraction to clarify the tissue distribution of the vaccine.

### IFN-γ ELISpot assays

The spleens of mice in each group were ground with a syringe and filtered through a 70-μm filter, and splenocytes were collected by centrifugation (300*g*) for 5 min. Erythrocytes were lysed with erythrocyte lysing solution for 5 min and terminated with serum-containing medium. Splenocytes were collected by filtration through a 40-μm filter and were counted with a cell counter. Mouse IFN-γ ELISpot PLUS Kit (ALP) plates (Mabtech, 3321-4APT-10) were washed thoroughly five times with PBS before use. The plates were blocked with RPMI-1640 medium containing 10% FBS for 0.5 hours at 37°C. Next, 2.5× 10^5^ freshly isolated splenocytes were incubated in the plate in the presence of neoantigen peptide (2 μg/ml) in RPMI-1640 medium containing 10% FBS for 18 hours at 37°C. Negative fractions were suspended in RPMI-1640 (Thermo Fisher Scientific) containing 10% FBS. Positive fractions were stimulated by 10 μl of phorbol 12-myristate 13-acetate + ionomycin (Dakewei, 2030421).

### T cell depletion

To evaluate the role of CD4^+^/CD8^+^ T cells during LPP-mRNA vaccine immunization, 100 μg of anti-mouse CD4 (BioXcell, clone GK1.5, BE003) or anti-mouse CD8 (BioXcell, clone 2.43, BE0061) with 100 μl of PBS were injected intraperitoneally (i.p.) on days −3, 0, 3, and 7. An equal amount of isotype control (BioXcell, clone LTF-2, BE0090) was injected as a control. Tumor cells were implanted at day 0, and the mice were immunized with LPP-mRNA vaccine twice a week from day 3 and euthanized on day 18.

### Cytotoxic lymphocyte killing test

After obtaining splenocytes from each group of mice, they were washed twice with PBS. Then, 2.5 × 10^6^ splenocytes were planted in the 24-well plates, and recombinant mouse IL-2 (10 ng/ml) was added to the medium to stimulate splenocytes for 1 week. Labeling of tumor cells (CT26-luc and MC38 cells) with CellTracker Deep Red (Thermo Fisher Scientific, C34565) was performed at a concentration of 1:1000 for half an hour, and the tumor cells were counted. Splenocytes were cocultured with the labeled tumor cells (CT26-luc and MC38 cells) at a ratio of 50:1 for 18 hours in a cell incubator. Live/Dead Fixable Aqua Dead Cell Stain Kit (Thermo Fisher Scientific, L34957) was used to differentiate between live and dead tumor cells, and the percentage of tumor cell death was detected by FACS.

### Western blot analysis

The 293T cells incubated with LPP-PCV for 24 hours were lysed following the manufacturer’s instructions, and protein concentration was measured using a bicinchoninic acid protein assay (Beyotime Biotech). Next, equal amounts of proteins were loaded onto a polyacrylamide gel, and electrophoresis was performed. The separated proteins were transferred from the gel to a polyvinylidene difluoride (PVDF) membrane using a transfer apparatus. The membrane was incubated in 5% milk in Tris-Buffered Saline-Tween 20 (TBS-T). The primary antibodies specific to the target protein were applied and incubated overnight at 4°C. MITD antibody was customized by ABclonal (1:1000), and β-actin was from Santa Cruz Biotechnology (1:2000, #sc-69879). The PVDF membrane was washed with TBS-T to remove unbound primary antibodies. Then, a secondary antibody was applied and incubated for 2 hours at room temperature. The membrane was washed in TBS-T, and then the luminescent solution was evenly covered. Last, the images were captured by a chemiluminescence imager.

### Animal model

Six- to eight-week-old C57BL/6 and BALB/c female mice were purchased from Vital River (Beijing, China) and were acclimated for 1 week before the start of the experiment. All experiments in vivo were conducted in compliance with the guidelines of the Ethics Committee of Tongji University (Shanghai, China) (TJBB00723105). Synthetic tumor models were generated by inoculating 2.5 × 10^5^ tumor cells subcutaneously in the left flanks of mice. Tumor cells (2.5 × 10^5^) were injected intravenously into 6- to 8-week-old mice to establish a lung metastasis model. The mice were randomly assigned to the control and treatment groups 3 days after the tumor cell inoculation. LPP-mRNA vaccines were administrated every 3 days or twice a week by two-point subcutaneous injection at the abdominal site 3 days after tumor inoculation. A total of four doses of the LPP-mRNA vaccine were administrated. Tumor growth and body weight were monitored every 3 days or twice a week. The following equation calculated the tumor volume

Tumor volume=1/2×Length×Width×Width

The mice were euthanized when the tumor volume reached to 2000 mm^3^, and the weight of tumor tissues was recorded.

### Clinical study

The clinical trial ChiCTR2100050688 was registered on the Chinese Clinical Trial Registry and was in compliance with the guidelines of the Ethics Committee of Shanghai East Hospital. The clinical trial was performed according to the Declaration of Helsinki. All patients provided written informed consent. Eligible patients were diagnosed as having malignant solid tumors and had tissue collected for further WES and RNA-seq. The patients were over 18 years of age, and there was no gender requirement. The physical fitness score on the ECOG scale was 0–1, and their expected survival period was over 6 months. According to the definition of Response Evaluation Criteria in Solid Tumors (RECIST) 1.1, there were measurable lesions. Laboratory inspections meet the following standards: (i) Bone marrow function: absolute blood neutrophil count (ANC) ≥ 1 × 10^9^/liter, hemoglobin ≥8 g/dl PLT ≥ 75 × 10^9^/liter; (ii) liver function: serum total bilirubin (STB), conjugated bilirubin (CB) ≤ upper limit of normal (ULN) × 1.5, ALT, AST ≤ ULN × 2.5 (in the absence of liver metastases), or ≤ ULN × 5 (in the presence of liver metastases); (iii) renal function: serum creatinine (Cr) ≤ ULN × 1.5, endogenous creatinine clearance (Ccr) ≥ 50 ml/min (calculated by Cockcroft-Gault formula). Exclusion criteria included: (i) participation in a clinical trial within 4 weeks; (ii) active infection; (iii) pregnant and lactating women; (iv) another active or progressive malignant tumors; (v) uncontrolled symptomatic brain metastases; (vi) use of immunosuppressive drugs; (vii) HIV, HBV, and HCV infections; (viii) severe autoimmune disease; (ix) history of organ transplantation, known bone marrow or hematopoietic stem cell transplantation; (x) those with abnormal coagulation function; (xi) uncontrolled or severe cardiovascular disease, diabetes, or major heart disease within 30 days.

Each patient considered for the study received detailed information on the study procedures, the experimental treatment, the potential risks and benefits of the treatment, possible standard treatment alternatives, and patient’s fundamental rights included in an experimental study or clinical trial. Patients may continue to use standard treatment for the disease during vaccine preparation and administration. After vaccine preparation, the vaccine was administered every 3 weeks by subcutaneous injection at 400 μg per dose until eight full doses had been used. Observations were made on the safety of the drug, monitoring the immune response and the clinical response of the tumor. The end points of observation were patient tumor progression, occurrence of serious adverse events, or patient withdrawal.

### Statistical analysis

All results are presented as means ± SD. Statistics were assessed with a one-way analysis of variance (ANOVA) test using Tukey’s correction for multiple group comparison and an unpaired two-tailed *t* test for two group comparison. Data were analyzed with the GraphPad Prism v8.0.2 software. *P* < 0.05 was considered statistically significant (*P < 0.05; **P < 0.01; ***P < 0.005; ****P < 0.001).
