# Supplementary material for: Lipopolyplex-formulated mRNA cancer vaccine elicits strong neoantigen-specific T cell responses and antitumor activity
Source: Sci Adv. 2024 Oct 11;10(41):eadn9961. doi: 10.1126/sciadv.adn9961 (PMC11468906; doi:10.1126/sciadv.adn9961)
Supplement: Supplementary file 1 — Figs. S1 to S13 Table S1 [file sciadv.adn9961_sm.pdf]

Science Advances
AAAS

Supplementary Materials for

Lipopolyplex-formulated mRNA cancer vaccine elicits strong neoantigen-specific T cell responses and antitumor activity

Ting Fan *et al.*

Corresponding author: Dean G Tang, dean.tang@roswellpark.org; Hangwen Li, lihangwen@stemirna.com; Chunyan Dong, cy_dong@tongji.edu.cn

Sci. Adv. **10**, eadn9961 (2024)
DOI: 10.1126/sciadv.adn9961

This PDF file includes:

Figs. S1 to S13
Table S1

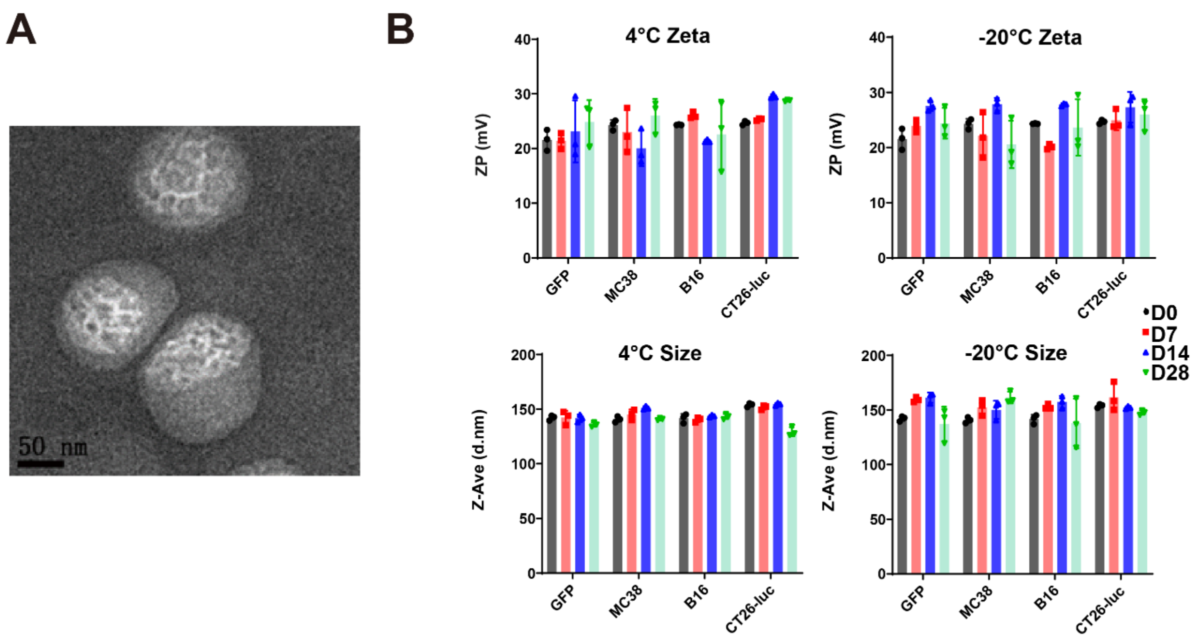

Figure S1. Characterizations of LPP-mRNA vaccine.

(A). Representative transmission electronic microscopy (TEM) image of LPP-mRNA vaccine particles.
(B). Time-dependent changes in particle size and zeta potential at 4°C and -20°C.

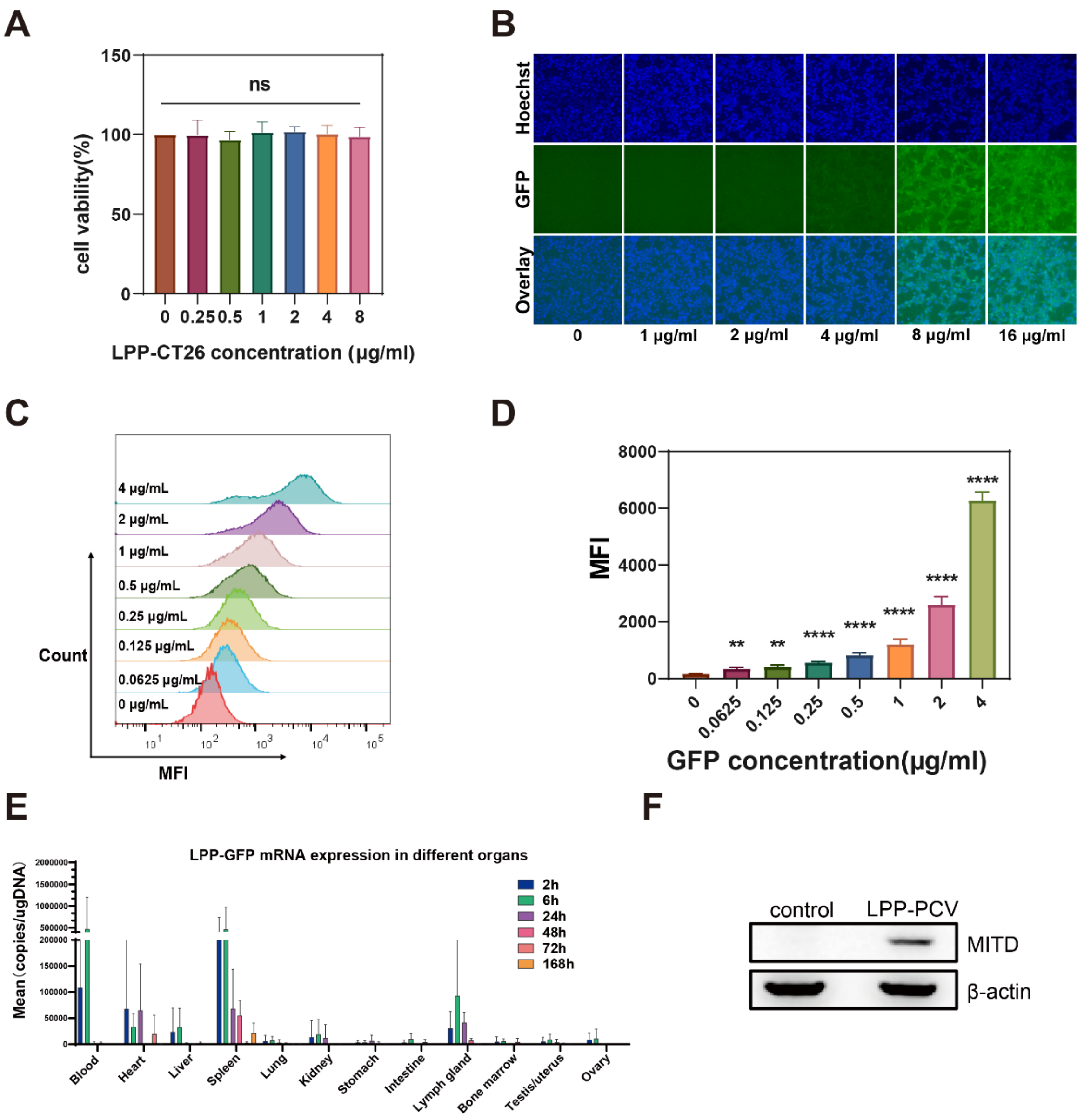

Figure S2. Figure S2. LPP-mRNA vaccine's translation efficiency is assessed both *in vitro* and *in vivo*.

(A). CCK8 detects cytotoxicity of LPP-CT26 on 293T cells at different concentrations. **(B)**. Fluorescence microscopy to detect the expression level of GFP after incubation of different concentrations of LPP-GFP with 293T cells for 24 hours. **(C-D)**. FACS detection of GFP expression after incubation of different concentrations of LPP-GFP with 293T cells for 24 hours. **(E)**. Distribution of LPP-GFP mRNA in tissues at 2 h, 6 h, 24 h, 48 h, 72 h and 168 h after LPP-GFP immunization

measured by RT-PCR. n=6 for each tissue. **(F)**. The LPP-mRNA vaccine was transfected into 293T cells, and the cells were harvested after 24 h. The levels of MITD in cell lysates were detected by Western blotting. Data were presented as mean ± SD. One-way ANOVA statistical test was performed for all data analysis (*$p < 0.05$, **$p < 0.01$, ***$p < 0.001$, ****$p < 0.0001$).

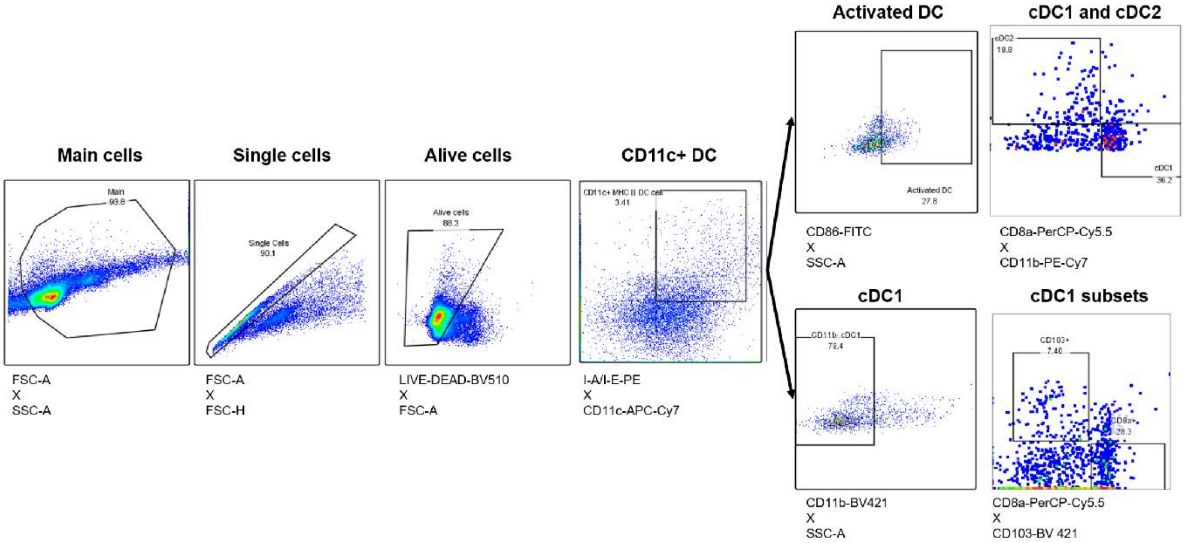

Figure S3. FACS gating strategy.

FACS gating strategy for differentiating DC cell subsets after immunization with LPP-mRNA vaccine.

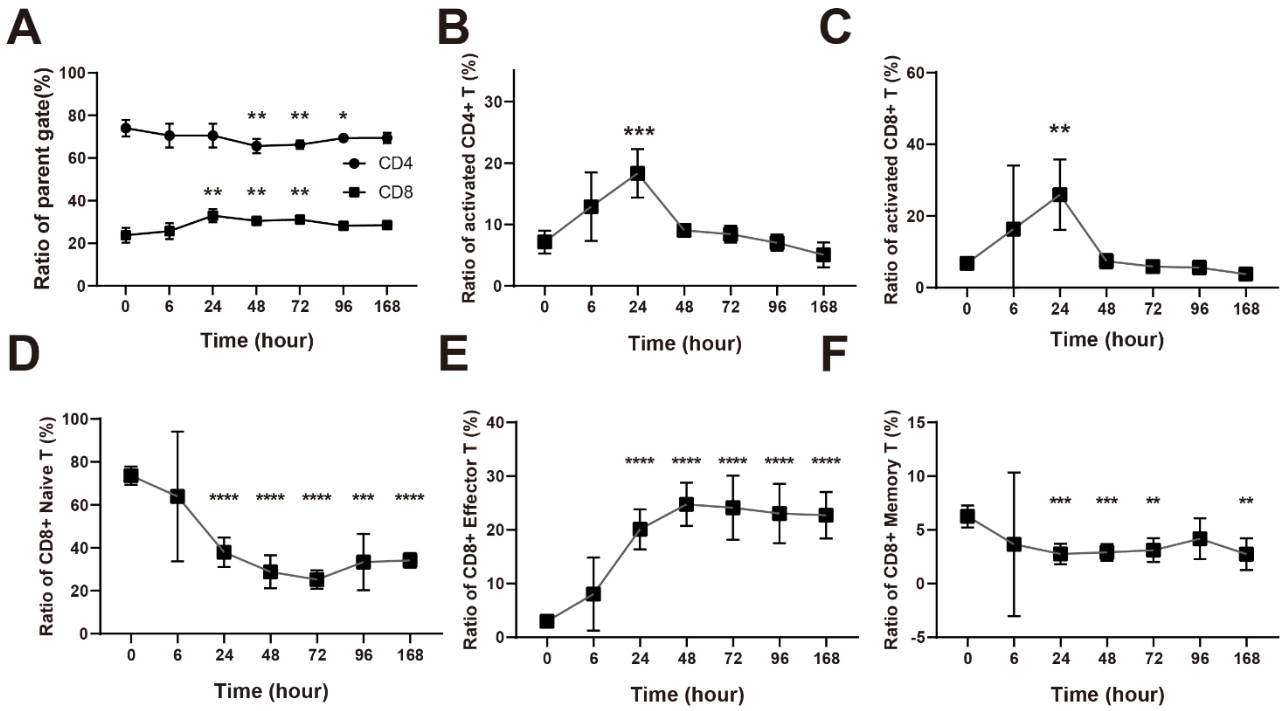

Figure S4. LPP-mRNA vaccine activated T cells subsets in the spleen *in vivo*.

(A). Proportion of CD4+ and CD8+ T in the spleen at different time point after LPP-mRNA vaccination. **(B-C)**. Proportion of CD4+ and CD8+ T activation in the spleen after LPP-mRNA vaccination. **(D-F)**. Content of naive T, effector T and memory T cells in the spleen at different time points. Data were presented as mean ± SD. One-way ANNOVA statistical test was performed was performed for all data analysis. T test (*$p < 0.05$, **$p < 0.01$, ***$p < 0.001$, ****$p < 0.0001$).

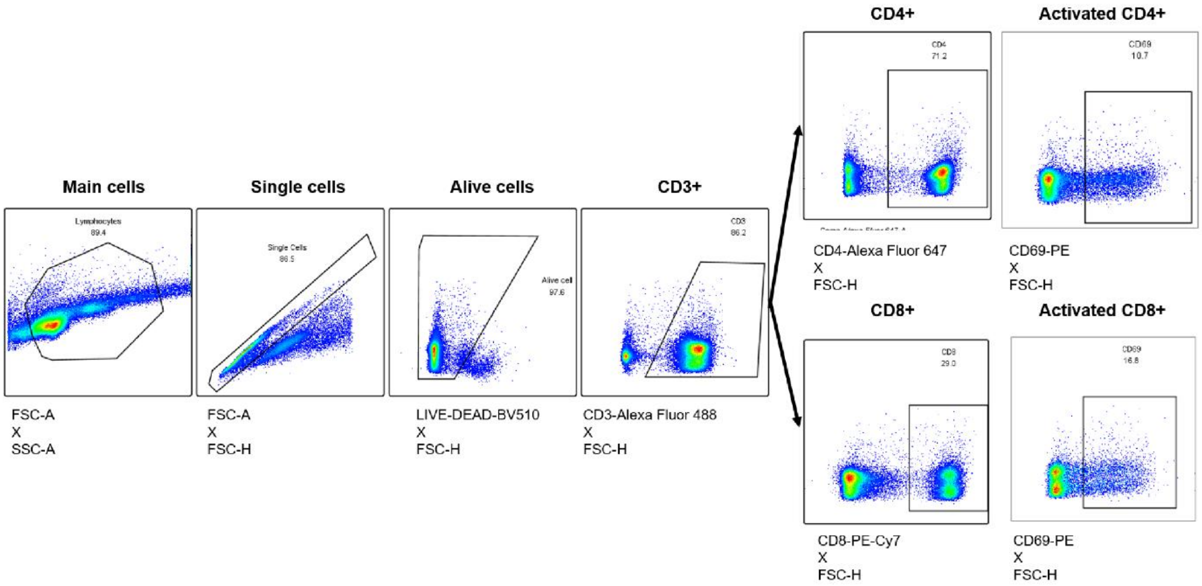

Figure S5. FACS gating strategy.

FACS gating strategy for differentiating activated T cell subsets after immunization with LPP-mRNA vaccine.

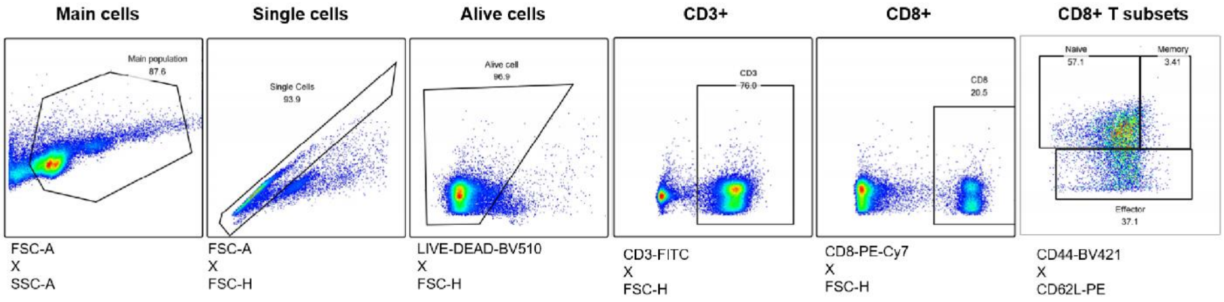

Figure S6. FACS gating strategy.

Flow analysis of T cell subsets in mice after immunization with LPP-mRNA vaccine. The figure illustrates the FACS gating strategy for various T cell subsets.

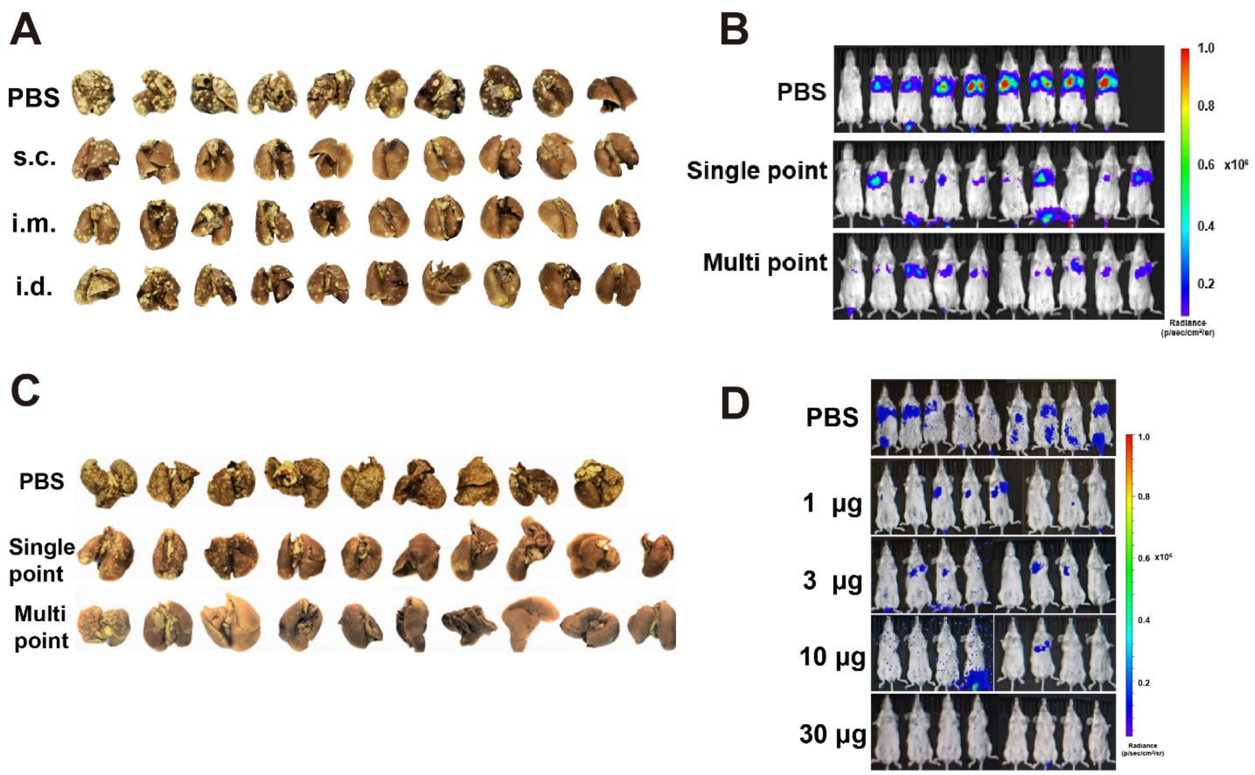

Figure S7. Selection of vaccination procedures for optimal tumor suppression efficiency.

(A). Image of CT26-luc lung metastasis on D18 after administering LPP-CT26 via 4 different routes. **(B)**. The IVIS image of CT26-luc bearing mice on Day 10. **(C)**. The CT26-luc lung metastasis picture on Day 18. **(D)**. The IVIS image of CT26-luc bearing mice with different LPP-CT26 dosage immunization on Day 14.

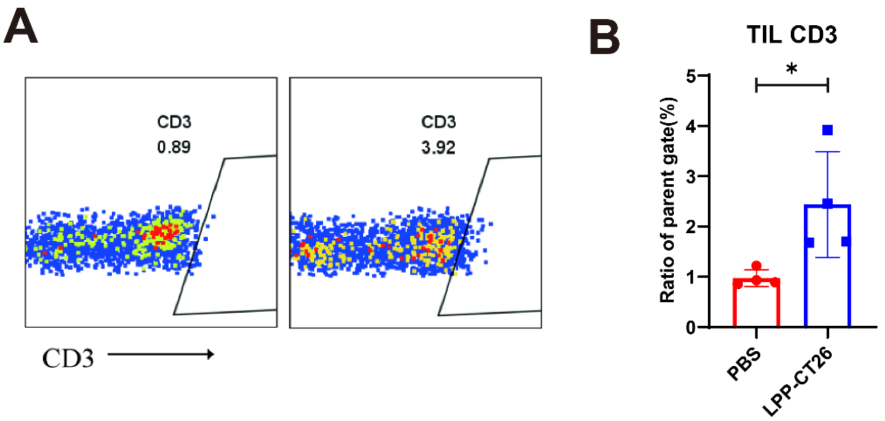

Figure S8. Proportion of TILs in CT26 subcutaneous synthetic tumor model.

(A). Representative images of TILs in the CT26 subcutaneous graft tumor model by FACS after LPP-CT26 immunization. **(B)**. Statistical analysis of the proportion of TILs in CT26 subcutaneous transplantation tumors. Data were presented as mean ± SD. One-way ANOVA statistical test was performed was performed for all data analysis. (*$p < 0.05$, **$p < 0.01$, ***$p < 0.001$, ****$p < 0.0001$).

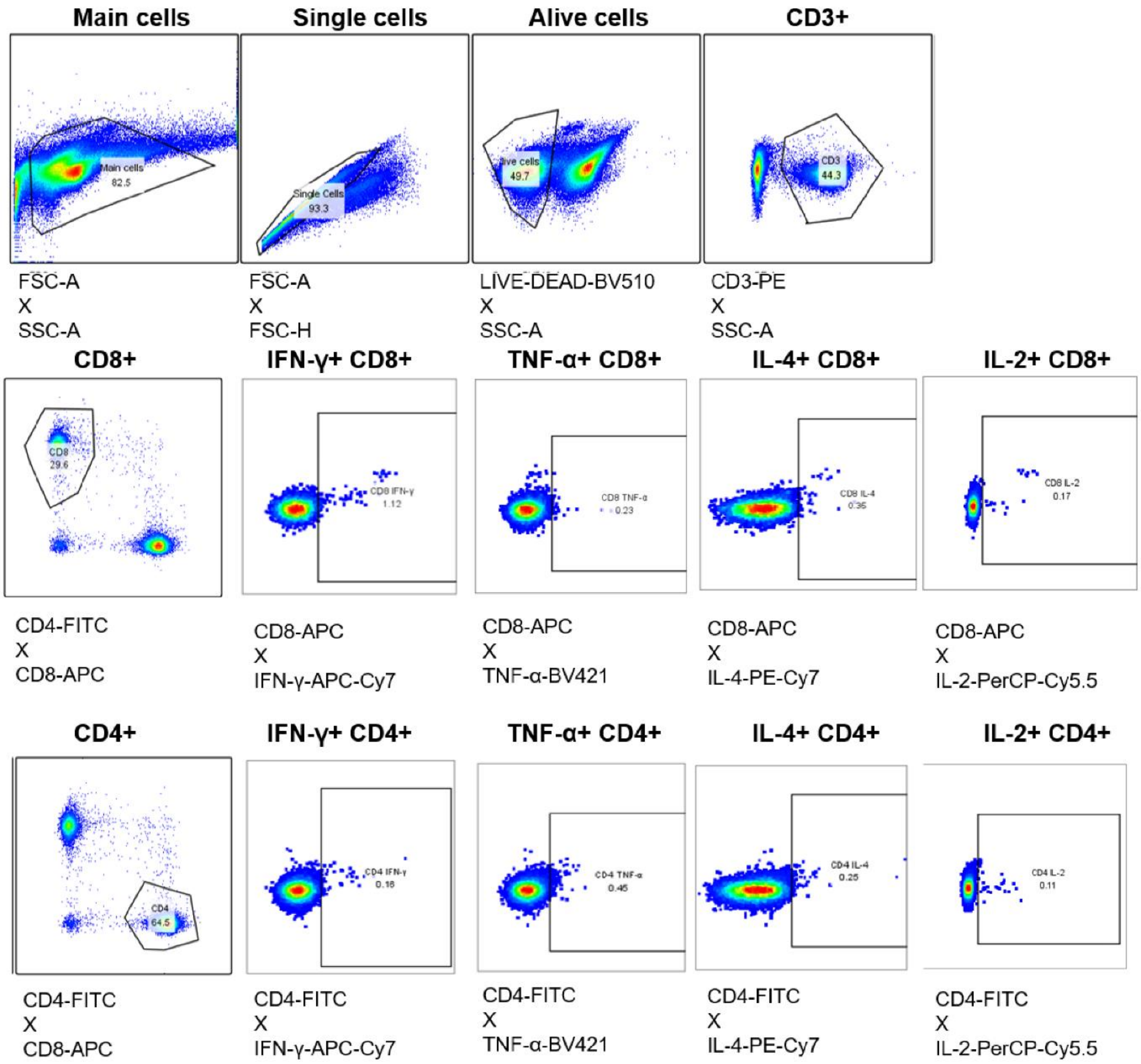

Figure S9. FACS gating strategy.

FACS gating strategy for ICS (intracellular cytokine staining) of different cytokines secreted by T cells.

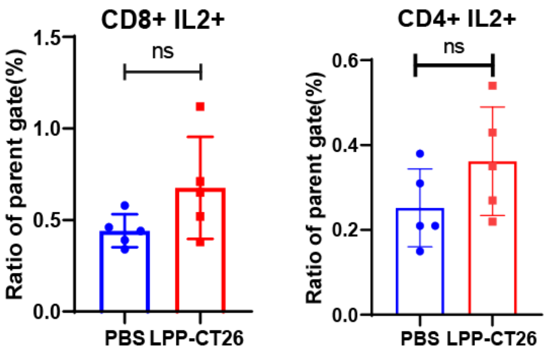

Figure S10. Intracellular cytokine staining of IL-2 by FACS in CD4+ or CD8+ T cells in primary splenocytes from mice treated with LPP-CT26 or PBS.

Data were presented as mean ± SD. One-way ANOVA statistical test was performed for all data analysis (*$p < 0.05$, **$p < 0.01$, ***$p < 0.001$, ****$p < 0.0001$).

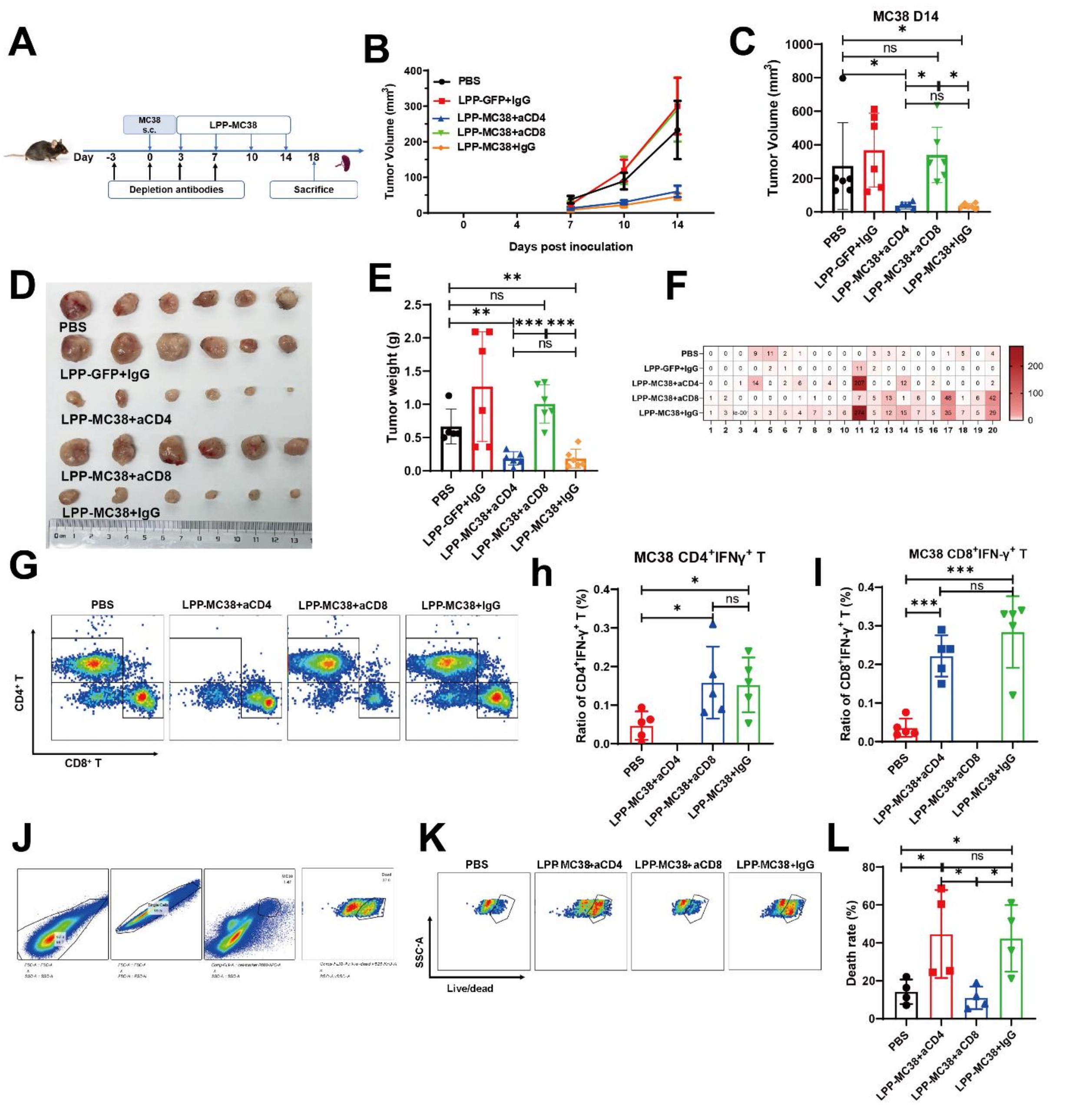

Figure S11. Anti-tumor efficacy of LPP-mRNA vaccine is dependent on CD8+ T cells in MC38 syngeneic tumor model.

(A). Schema for *in vivo* CD4+/CD8+ T cell depletion experiments. Groups of n=6 C57BL/6 mice were administrated with T cell depletion antibody via i.p injection. MC38 syngeneic tumors were established by s.c injection of MC38 cells on Day 0 and immunization with LPP-MC38 was started from Day 3. **(B)**. Growth curve of MC38 tumors in mice immunized with LPP-MC38 after T cell depletion. **(C-E)**. Tumor vol. of MC38 model on Day 14 (C), and endpoint MC38 tumor image (D) and weight (E) on

Day 18. **(F)**. IFN-γ ELISpot assay of splenocytes in MC38-bearing mice. **(G)**. Representative FACS plot images of T cell subsets in mouse splenocytes after T cell depletion. **(H-I)**. ICS by FACS in CD4+/CD8+ T cells in mouse bulk splenocytes after T cell depletion. **(J)**. FACS gating strategy for detection of tumor cell death after co-culturing MC38 tumor cells with T cells. **(K)**. Representative FACS plot images of MC38 cell death. **(L)**. Percentage of MC38 cells killed after co-culture with T cells *in vitro* via FACS assay. Data were presented as mean ± SD. One-way ANOVA statistical test was performed for all data analysis (*$p < 0.05$, **$p < 0.01$, ***$p < 0.001$, ****$p < 0.0001$).

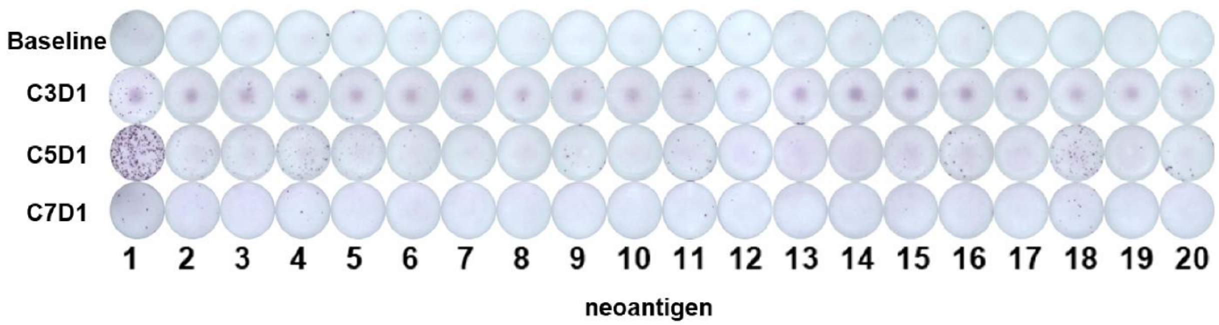

Figure S12. ***Ex vivo*** **IFN-γ ELISpot of PBMCs from patient 05002 was performed with neoantigens at different time points.**

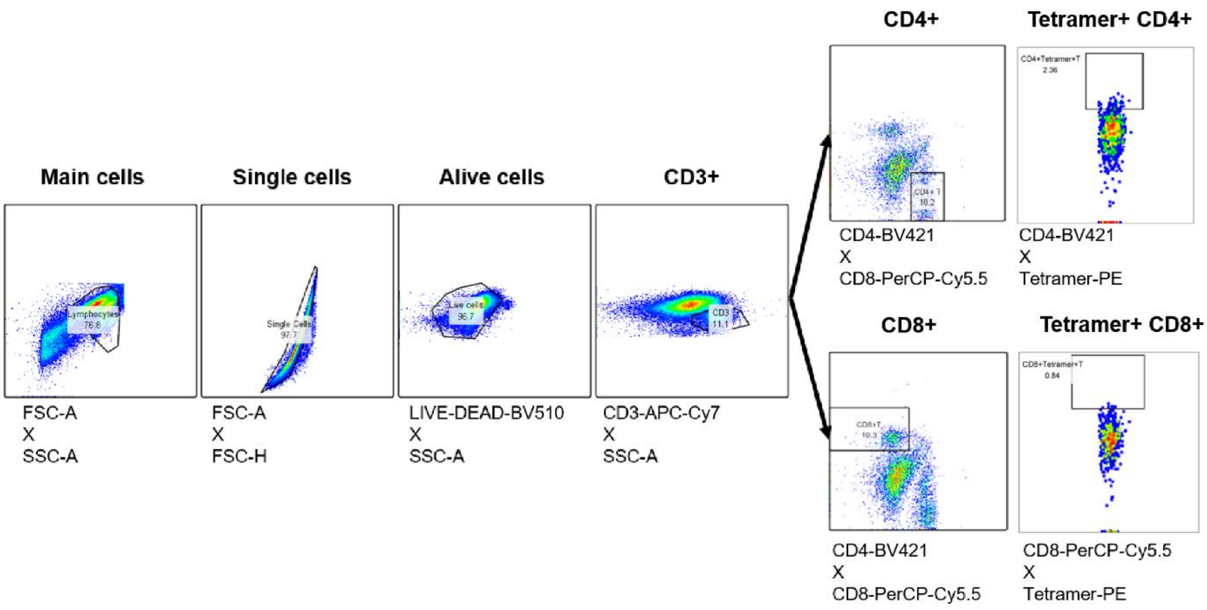

Figure S13. FACS gating strategy.

The PBMC from the patients immunized with LPP-PCV were stained with MHC tetramer, and the proportion of positive MHC tetramer was quantified by FACS.

Table S1. Overview of the neoepitopes used for *in vivo* studies and *ex vivo* analyses targeting CT26-luc, MC38 and B16F10 cells. Bold amino acid denotes the position of the point mutation. Underlined amino acids show the best predicted MHC neoepitope.

	Gene	Substitution	27AA sequence used for immunization	MHC_type	Vaccine
1	Anapc1	D241N	GSLFGSS*RVQYVV**N**PAVKIVFL*NIDPS	MHC-I	LPP-CT26
2	Mkrn1	E352K	NFVI*PSEYWVEEK**K**EKQKL*IQKYKEAM	MHC-II	
3	Gatad2b	N196I	LLKKLR*QSQLQKE**I**VVQKTPV*VQNAAS	MHC-II	
4	Ddx27	S297F	ITTCLAVGGL*DVK**F**QEAALRAAPDI*LI	MHC-II	
5	Vps26c	G148R	KT*CEFIVHSAPQK**R**KLT*PSPVDFTITP	MHC-II	
6	Aldh18a1	P154S	LHSG*QNHLKEMAI**S**VLEAR*ACAAAGQS	MHC-II	
7	Uck2	P247L	SKRQTNGY*LNGYT**L**SRKRQASES*SSRP	MHC-I	
8	Eif2a	L485F	KPHPGSDKPLS*KTA**F**KNQRKHEAKKA*A	MHC-I	
9	Abhd4	G330A	QRP*DSYVRDMEIE**A**ASHH*VYADQPHIF	MHC-II	
10	Aprt	A47P	KDPDSF*RASIRLL**P**SHLKSTH*SGKIDY	MHC-II	
11	Snx5	T341A	KAR*LKSKDVKLAE**A**HQQE*CCQKFEQLS	MHC-II	
12	Nap1l4	V63I	HTPSSYIETL*PKA**I**KRRINALKQLQ*VR	MHC-I	
13	Stat6	M703I	PQSIHS*FQSLEES**I**SVLPSFQ*EPHLQM	MHC-I	
14	Rbm18	G91A	TK*QEAEQAIQCLN**A**KLA*LSKKLVVRWA	MHC-II	
15	Vps50	A357T	MEWHEKHD*NEETAA**T**AEGSNVMS*TEEA	MHC-II	
16	Rab3ip	C373R	LQP*IRFVKASAVE**R**GGPK*KCALTGQSK	MHC-II	
17	Ppip5k2	S952F	EVDRAVMLF*KPLV**F**EPIHIHRKSP*LPR	MHC-I	
18	Gadd45gip1	E144K	QMIENWRKQK*RERW**K**KIQADKERRA*RL	MHC-II	
19	Atrx	T782I	GSDFDTKKG*KSTE**I**SIISKKKRQN*YSE	MHC-II	
20	Trim14	F130V	RLLLD*EEEVLAKK**V**IDKSTQ*LTLQVYR	MHC-II	
1	Usp25	E360V	ESLHSDNSGKSG*Q**V**HWFTEL*PPVLTFE	MHC-I	LPP-MC38
2	Itgb2	Q412L	DCDG*VQINNPVTF**L***VKVMASECIQEQS	MHC-I	
3	N4bp2l2	R65L	NNRV*AVATINFRR**L***VCPQEDKTSTDVL	MHC-I	
4	Spire1	D40Y	GEKRSISAI*RSYQ**Y**VMKI*CAAHLPTES	MHC-I	
5	Klf12	P203L	VQSVP*VVYTAVRS**L***GNVNNTIVVPLLE	MHC-I	
6	4932438A13Rik	S4801I	KNSSLLSGFRG*GS**I**YNHETETI*FALPR	MHC-I	

7	Fanci	839.H/X	ESLSVLR*SSGEFM**TL**CSERHFAEDSAA	MHC-I	
8	Pi4kb	C212F	VHRCRQSIN*FSLQ**F**ALL*LGAYSSDMHI	MHC-I	
9	Slc25a54	346.G/X	AFSKGVPNLL***S**IIPYAG**L***DLTIFELLK	MHC-I	
10	Olfr854	Q286L	KTAVAS*VMYSVVP**L***MLNPFIYSLRNRD	MHC-I	
11	Olfr175	134.T/NX	DRYVAICNPLQYHQT***AMWPYAT***LCSTT	MHC-I	
12	Olfr828	104.T/X	TLFCSFESCLLSV***M**AYDRYVA**I***CHPLN	MHC-I	
13	Asb15	D428Y	DTRFPSAIQYA*LN**Y**EIMLRL*LLNNGYQ	MHC-I	
14	Alg8	F285V	FKRGLCHAYWAPN***V**WALYNAL*DKVLSV	MHC-I	
15	Sec16a	Q1085L	LERAQPEL*VPPRP**L**NSPQVPQAS*CPEP	MHC-II	
16	Tgfb2	L182F	ASNLVKAEFRVFRFQNPKARVAEQRIE	MHC-II	
17	Card6	G667V	VI*QDTQVSPRTIE**V**ENQ*QPQSQTKSPS	MHC-II	
18	Med12l	P2018Q	GVVLSPS*YNSRAY**Q**AAHSSPAL*MERLR	MHC-II	
19	Tgfbr3l	P229A	PSKSLPGR*SVHPE**A**PAPAPAALE*PAPV	MHC-II	
20	Noxo1	D248E	QFCTTQAYEGSRSEELSVPSGARVHVL	MHC-II	
1	Dync1h1	1600.E/X	EGWQTCWGRSRKHWG***STWNGSARL***SPG	MHC-I	
2	Tbcd	588.A/X	AHNLTPQVPEY***I**AMHVFPA**L***LLMTQSP	MHC-I	
3	Klhl26	E487A	PAADRWEP*R**A**PMRAPRVLHAM*LGAAGR	MHC-I	
4	Hipk3	S702F	QVTPMAPAA*ATLT**F**EGM*AGSQRLGDWG	MHC-I	
5	Piezo1	1991-1992.-/I	LMFLADIVDIIII***I**IFGFWAF**G***KHSAA	MHC-I	
6	Hsf2	K72N	ASFVRQLNM*YGFR**N**VVHI*ESGIIKQER	MHC-I	
7	Nckipsd	K492N	LARDMQTDTQDH*Q**N**LCYSAL*VLAMVFS	MHC-I	
8	Mfhas1	P622A	LLNHRLQILSPV*L**A**VSCRDPL*QLQRLR	MHC-I	
9	Aldh1b1	A26V	CLQGRT*TSYSTAA**V***LPNPIPNPEICYN	MHC-I	LPP-B16
10	Herc6	K661N	FPEKMSSPP*YLIL**N**VRRSHL*VEDTLRQ	MHC-I	
11	Arhgap28	A515V	LMV*MALPDANRDT**V***QALMAFFNKVIAN	MHC-I	
12	Il11	S103F	LPGVLTRLRVD*LM**F**YLRHV*QWLRRAGG	MHC-I	
13	Notch1	D680A	EPGYT*GSMCNVNI**A***ECAGSPCHNGGTC	MHC-I	
14	Shmt1	F119V	GVNVQPYSGSPAN***V**AVYTALV*EPHGRI	MHC-I	
15	Prob1	P811A	SPQACPN*SSLRAA**A**EL*ETPLVAPATAV	MHC-I	
16	Rpgrip1l	K818T	RASYLQP*HAYVVY**T**FF*DFPDHDTAIVP	MHC-I	

17	Mta1	P547L	LEAVL<u>*RYLETHPR**L***</u>PKPDPVKSSSSVL	MHC-I
18	Rab3gap1	D539Y	KKARDEGKK<u>*TSLS**Y**STT*</u>SAYPGDAGKT	MHC-I
19	Mlx	G23A	DPW<u>*VKASFADAHA**A**EGRA*</u>GRARARRGS	MHC-II
20	Mn1	F39I	AG<u>*LSMNAHFKAPA**I**HAG*</u>PPTGPVDPAI	MHC-II
